# Comparative genome analysis reveals high-level drug resistance markers in a clinical isolate of *Mycobacterium fortuitum* subsp*. fortuitum* MF GZ001

**DOI:** 10.3389/fcimb.2022.1056007

**Published:** 2023-01-04

**Authors:** Md Shah Alam, Ping Guan, Yuting Zhu, Sanshan Zeng, Xiange Fang, Shuai Wang, Buhari Yusuf, Jingran Zhang, Xirong Tian, Cuiting Fang, Yamin Gao, Mst Sumaia Khatun, Zhiyong Liu, H. M. Adnan Hameed, Yaoju Tan, Jinxing Hu, Jianxiong Liu, Tianyu Zhang

**Affiliations:** ^1^ State Key Laboratory of Respiratory Disease, Guangzhou Institutes of Biomedicine and Health, Chinese Academy of Sciences, Guangzhou, China; ^2^ Guangdong-Hong Kong-Macao Joint Laboratory of Respiratory Infectious Diseases, Guangzhou, China; ^3^ University of Chinese Academy of Sciences, Beijing, China; ^4^ China-New Zealand Joint Laboratory on Biomedicine and Health, Guangzhou, China; ^5^ State Key Laboratory of Respiratory Disease, Guangzhou Chest Hospital, Guangzhou, China; ^6^ School of Life Sciences, University of Science and Technology of China, Hefei, Anhui, China; ^7^ National Clinical Research Center for Infectious Diseases, Guangdong Provincial Clinical Research Center for Tuberculosis, Shenzhen Third People’s Hospital, Shenzhen, China

**Keywords:** *Mycobacterium fortuitum*, morphology, comparative genomic analysis, drug resistance, pathogenesis

## Abstract

**Introduction:**

Infections caused by non-tuberculosis mycobacteria are significantly worsening across the globe. M. fortuitum complex is a rapidly growing pathogenic species that is of clinical relevance to both humans and animals. This pathogen has the potential to create adverse effects on human healthcare.

**Methods:**

The MF GZ001 clinical strain was collected from the sputum of a 45-year-old male patient with a pulmonary infection. The morphological studies, comparative genomic analysis, and drug resistance profiles along with variants detection were performed in this study. In addition, comparative analysis of virulence genes led us to understand the pathogenicity of this organism.

**Results:**

Bacterial growth kinetics and morphology confirmed that MF GZ001 is a rapidly growing species with a rough morphotype. The MF GZ001 contains 6413573 bp genome size with 66.18 % high G+C content. MF GZ001 possesses a larger genome than other related mycobacteria and included 6156 protein-coding genes. Molecular phylogenetic tree, collinearity, and comparative genomic analysis suggested that MF GZ001 is a novel member of the M. fortuitum complex. We carried out the drug resistance profile analysis and found single nucleotide polymorphism (SNP) mutations in key drug resistance genes such as rpoB, katG, AAC(2')-Ib, gyrA, gyrB, embB, pncA, blaF, thyA, embC, embR, and iniA. In addition, the MF GZ001strain contains mutations in iniA, iniC, pncA, and ribD which conferred resistance to isoniazid, ethambutol, pyrazinamide, and para-aminosalicylic acid respectively, which are not frequently observed in rapidly growing mycobacteria. A wide variety of predicted putative potential virulence genes were found in MF GZ001, most of which are shared with well-recognized mycobacterial species with high pathogenic profiles such as M. tuberculosis and M. abscessus.

**Discussion:**

Our identified novel features of a pathogenic member of the M. fortuitum complex will provide the foundation for further investigation of mycobacterial pathogenicity and effective treatment.

## Introduction

Non-tuberculosis mycobacteria (NTM) are ubiquitous, free-living, environmental saprophytic organisms that can cause human infections, including respiratory and skin diseases in both immunocompetent and immunocompromised individuals (Chan and Iseman, 2013; Maya et al., 2022). The prevalence of NTM infections has risen over the years around the world (Reves and Schluger, 2014). The yearly incidence of lung infections caused by NTM has increased from 3.13 to 4.73 per 100000 people, whereas the prevalence of NTM rose dramatically from 4.24% in 2014 to 12.68% in 2021, indicating a significant increase in the NTM outbreak in China. (Winthrop et al., 2020; Jia et al., 2021; Sun et al., 2022). The majority of NTM are rapidly growing species that have been associated with serious infectious diseases (De Groote and Huitt, 2006). Importantly, previous investigations reported that *M. abscessus* could be transmitted from person to person, and its prevalence has been widely observed in hospitals (Aitken et al., 2012; Bryant et al., 2013). The fast-growing mycobacteria comprise some clinically relevant species which include *M. fortuitum*, *M. abscessus*, *M. smegmatis*, and *M. chelonae* (De Groote and Huitt, 2006). *M. fortuitum* is frequently isolated from both respiratory and non-respiratory specimens (Park et al., 2008; Bryant et al., 2013).

*M. fortuitum* complex comprises opportunistic pathogens usually found in water, soil, and dust that are of clinical relevance to both humans and animals (Pavlik et al., 2021). The *M. fortuitum* complex includes *M. fortuitum*, *M. peregrinum*, *M. mageritense, M. porcinum*, *M. septicum*, *M. conceptionense*, *M. boenickei*, *M. houstonense*, *M. brisbanense*, *M. farcinogenes, M. senegalense*, and *M. setense* (Johansen and kremer, 2020; Brown-Elliott and Philley, 2017; Brown-Elliott and Wallace, 2002). *M. fortuitum* is a rapidly growing human-pathogenic species that cause pulmonary, eye, post-surgical, and catheter as well as skin and soft tissue infections (Koh et al., 2006; Brown-Elliott et al., 2012; Diaz et al., 2019; Erber et al., 2020). *M. fortuitum* in respiratory samples has been categorized based on colony morphologies, growth characteristics, and *in vitro* resistance to anti-mycobacterials (Daley and Griffith, 2002).

Whole-genome sequencing (WGS) technologies are the new strategies for understanding the molecular basis of drug resistance, metabolism, and evolution of pathogens. The conventional methodologies may have several drawbacks, particularly discordance between phenotypic and genotypic susceptibility testing outcomes. Illumina HiSeq sequencing can provide a variety of sequencing data for differentiating the distinct variable gene expressions between various samples. The sequencing data can also help in the assembly of *de novo* organism genomes (Austin et al., 2017). Highly accurate base-by-base sequencing is provided by this technique with almost no errors and up to 750 GB of data can be produced per sequencing run (Teng et al., 2017). Despite these advantages, Illumina sequencing’s low-quality transcripts and short reads can significantly reduce the scope of analyses of transcriptional variations and accurate annotation (Hert et al., 2008). However, WGS employs a short-read sequencing platform that allows for the identification of additional resistance-associated mutations. For instance, due to the repetitive structure and high GC content of the mycobacterial genome, amplification bias occurs frequently during library preparation, resulting in fragmented genome assembly and other genetic variations such as INDEL and copy number variations (Treangen and Salzberg, 2011; Leung et al., 2017).

The recently developed PacBio RS II with single-molecule real-time (SMRT) sequencing methods overcome the drawbacks of conventional methods. PacBio RS II offers long-read or whole transcriptomes (Eid et al., 2009) which enable the large-scale long-read transcript collection with complete coding sequences and the characterization of the various gene families. Moreover, SMRT has enabled *de novo* assembly of the mycobacterial genome much easier (Roberts et al., 2013). SMRT sequencing can easily span highly repetitive DNA sequences due to its average read length of 10–20 kbp (Qi et al., 2013; Zhu et al., 2016; Ferrarini et al., 2013). Additionally, it can reduce the number of gaps in the final assembled mycobacterial genome. Isolation of new rapidly growing mycobacteria (RGM) species and comprehensive analysis of the WGS of several mycobacterial isolates seem to be important for such kind of research.

In this study, we sequenced *M. fortuitum* subsp*. fortuitum* (designated as MF GZ001) isolated from a patient with pulmonary infection. This was deduced from conserved sequences of *16S rRNA hsp65*, and *rpoB* genes. We performed comparative studies with RGM and slow-growing mycobacteria (SGM) to understand the genomics, phylogeny, pathogenicity of this mycobacterial species, and the evolution of drug resistance. This investigation provides a genome-based description, which is ensuring that it’s related to the *M. fortuitum* complex and displayed novel features of a potential pathogenic species.

## Materials and methods

### Strains collection and growth conditions

The MF GZ001 clinical strain was collected from the sputum of a 45-year-old male patient with a pulmonary infection. The patient with the symptoms of non-paroxysmal irritant cough with yellow and white sputum, chest pain, shortness of breath, headache, and low fever was admitted to the Guangzhou Chest Hospital, Guangzhou, China. The isolate was grown on Middlebrook 7H11 agar (Becton, Dickinson, and Company) supplemented with 0.2% glycerol (Shanghai Macklin Biochemical, Shanghai, China) and 10% oleic acid-albumin-dextrose-catalase (OADC) and in Middlebrook 7H9 (Difco, Becton, Dickinson and Company, New Jersey, USA) broth medium supplemented with 10% OADC, (Difco) and 0.05% Tween-80 (Amresco, USA).

### Growth kinetics and morphology detection

To determine the bacterial growth curves, three mycobacterial strains MF GZ001, *M. abscessus* GZ002 (Guo et al., 2016; Chhotaray et al., 2020), and *M. smegmatis* C^2^ 155 were collected from Guangzhou Chest Hospital and performed phenotypic characterization. Firstly, the bacterial strains were grown in Middlebrooks 7H9 broth medium to log phase. Then the strains were diluted up to OD_600_ 0.01 in each 100 mL flask of every strain and placed in a shaking incubator for 72 hrs at 200 rmp to determine the bacterial growth curve. The bacterial growth rates were measured by detecting OD_600_ readings of bacterial strains every 6 hrs intervals using a spectrophotometer. The bacterial growth curve analysis was performed by GraphPad Prism version (8.0.2).

For the colony morphology study, the mycobacterial strains were cultured in Middlebrook 7H9 broth medium up to OD_600_ 1. Then all bacterial strains were equalized by dilution at 10^-5^ and cultured for 7 days at 37 *^°^* C. Bacterial colony images were captured after 7 days of incubation and visualized by microscope (OLYMPUS TH4-200) to determine the size and surface structure of the colony respectively.

### Drug susceptibility testing (DST)

DST was performed by using broth micro-dilution methods according to the European Committee on Antimicrobial Susceptibility Testing (EUCAST) guidelines (Krishnan et al., 2009; Schon et al., 2020). The strains were grown in Middlebrook 7H9 broth medium (Difco, Becton, Dickinson and Company, New Jersey, USA) supplemented with 10% OADC, (Difco), 0.2% glycerol and 0.05% Tween-80 (Amresco, USA) for initial culture. Later, Middlebrook 7H9 (final pH 6.6 ± 0.2) broth medium supplemented with 10% OADC, 0.2% glycerol without Tween-80 was used in the preparation of inocula for DST. Based on EUCAST guidelines the inocula were fixed up to OD_600_ 0.6 with a final concentration of 1 × 10^5^ to 5 × 10^5^ CFU/mL. Cells in 96 well plates were incubated at 37°C. Several anti-microbials including rifabutin, imipenem, rifampicin (RIF), vancomycin, streptomycin, amikacin, clarithromycin, ethambutol, isoniazid, sulfamethoxazole, linezolid, and clofazimine were used to perform DST (**Table S11**). The MIC was defined based on the EUCAST formula as the lowest concentration of drugs that inhibited visible mycobacterial growth in wells.

### Library construction and WGS

MF GZ001 strain was cultured in 7H9 broth and extracted the genomic DNA (gDNA) by using MAagNA Pure LC DNA Isolation Kit III. The gDNA was fragmented and collected for the preparation of SMRTbell DNA template libraries. Briefly, the DNA fragments were end-repaired and the barcode overhang adapter-ligated by removing the single-strand overhangs. The library was quantified using a Qubit (version 3.0) Fluorometer (Invitrogen, Carlsbad, CA), and checked the library size using an Agilent 2100 Bioanalyzer System. Subsequent steps were followed as per the manufacturer’s instructions to prepare the SMRTbell library. The constructed library was sequenced using the Sequel II sequencing platform and PacBio reads were assembled using HGAP4 (version 4.0)/Falcon of WGS-Assembler (Version 8.2) (Myers et al., 2000; McCarthy, 2010). The genome sequence was re-corrected with Pilon software using previous Illumina data or Quiver using Pacbio reads to resolve the errors that were found during SMRT sequencing. The paired-end library was constructed by using the Illumina HiSeq instrument (Illumina, San Diego, CA, USA). The library construction protocol and bioinformatics analysis are broadly illustrated in the **supplementary material** (**Supplementary material 1**). The preliminary Illumina raw data reads were trimmed at the percentage of bases with a Phred value greater than 20 or 30 (less than 0.1%/1% probability of error).

The unnecessary bases and reads of Pass Filter data were removed to get clean data using Cutadapt (version1.9.1) and the Burrows-Wheeler Alignment tool (BWA) (version 0.7.12) (Li and Durbin, 2009) was used to align the clean data generated from MF GZ001 to *M. fortuitum* CT6 reference complete genome sequence (NZ_CP011269.1) (Costa et al., 2015). All statistical analyses of raw data which were obtained from Illumina and SMRT sequencing are attached in the **supplementary materials** ((**Tables S1–S6**). The Prodigal (version 3.0.2, prokaryote) (Delcher et al., 2007) and Augustus (version 3.3, eukaryotes) (Stanke et al., 2006) both gene-finding software were used for predicting the coding genes. Non-coding RNA (ncRNA) includes rRNA, tRNA, snRNA, snoRNA and microRNA. Among the ncRNA, tRNAs and rRNA were detected by using tRNAscan-SE (version 1.3.1) (Lowe and Eddy, 1997), and Barrnap (version 0.9) respectively in the genome assembly. Additionally mapping Rfam (version 12.2) (Nawrocki et al., 2015) method was used to predict other ncRNAs. The coding genes were annotated with National Center for Biotechnology Information (NCBI) NR database by Diamond blastp. Functional categories in the genome were assigned to the Gene Ontology (GO) by using InterProScan software (Harris et al., 2004), to the Clusters of Orthologous Groups of proteins (COGs/KOG) (Tatusov et al., 2003; Han et al., 2018) database using rpstblastn software, and to the Kyoto Encyclopedia of Genes and Genomes (KEGG) (Kanehisa and Goto, 2000) pathway database by performing the KEGG database (http://www.genome.jp/kegg/) with Blastn software. Carbohydrate-Active Enzyme (CAZy) annotation was displayed using Diamond blastp (Luo et al., 2022). The Pfam was annotated for a large collection of protein families using the Pfam database (http://pfam.xfam.org/) (Punta et al., 2012), and Swiss_Prot was annotated by applying the Swiss_Prot database (https://www.ebi.ac.uk/uniprot) (Magrane, 2011). The Circos (version 0.69) software was used to display the circular plot and to describe the common feature of the genome.

### Construction of phylogenetic trees

The evolutionary trees were constructed to represent the proximity of the relatedness between referred genome MF GZ001 and other 30 mycobacterial species. Single and combined gene-based (*16S rRNA*, *hsp65*, and *hsp65-rpoB*) three phylogenetic trees were constructed using FastTree and Mafft software (Price et al., 2010; Katoh and Standley, 2013; Robbertse et al., 2017). To generate the phylogenetic trees, the gene sequences were aligned with the reference sequences using Mafft software (version 7.310) (Katoh and Standley, 2013), and then evolutionary trees were constructed by using FastTree (Version 2.1.10.Dbl) (Price et al., 2010), which predicts nucleotide evolution using the Jukes-Cantor model and infers phylogenetic trees *via* approximately maximum-likelihood methods. Moreover, a comparison of the genetic relatedness between prokaryotic organisms is performed using average nucleotide identity (ANI) analysis. The online ANI tool was used to calculate the ANI value of the MF GZ001 genome as well as of its closely related species (https://www.ezbiocloud.net/tools/ani) (Yoon et al., 2017).

### Comparative genomic analysis

#### SNP/INDEL detection and annotation

SNP/INDEL was performed based on the result of MUMmer alignment between assembled genome sequence of MF GZ001 and the reference genomic sequences of *M. fortuitum* CT6 (NZ_CP011269.1), *M. abscessus* GZ002 (NZ_CP034181.1) (Chhotaray et al., 2020), *M. smegmatis* C^2^ 155 (NZ_CP054795.1), and *M. tuberculosis* H37Rv (NC_000962.3). Annovar software (Yang and Wang, 2015; Wang, 2020) was used to understand the annotation of mutation sites, the construction of the SNP/indel distribution map, and the detection of amino acid change caused by mutations.

#### Collinearity analysis

The study of multiple genome alignments in the context of identified genome MF GZ001 and reference genome *M. fortuitum* strain CT6 (CP011269.1), *M. abscessus* GZ002 (NZ_CP034181.1), *M. smegmatis* C^2^ 155 (NZ_CP054795.1), and *M. tuberculosis* H37Rv (NC_000962.3) collinearity relationship was constructed using MAUVE software version 2.4.0 (Huang et al., 2022). For the collinearity analysis, all reference genomes were downloaded from the NCBI database.

#### Core and pan genes analysis

*M. fortuitum* complex strains, including MF GZ001, other SGM, and its closely related strains for example *M. fortuitum* CT6 (CP011269.1), *M. brisbanense* UM_WWY (NZ_BCSX00000000.1), *M. septicum* DSM44393 (NZ_CP070349.1), *M. alvei* CIP103464 (NZ_AP022565.1) and *M. mageritense* (NZ_AP022567.1) were conducted to identify the core, strain-specific genes, clusters, and uncharacterized genes by ORTHOMCL (version 1.4). Using ORTHOMCL, the annotated protein sequences from the assemblies MF GZ001, SGM, and RGM were grouped into orthologous families. The ortholog clustering output of the OrthoMCL analysis was converted into an ortholog matrix and visualized as a Venn diagram (Wang et al., 2017).

#### Drug-resistant genes and virulence factors distribution, prediction, and analysis

Drug-resistant gene prediction and distribution were done using the comprehensive antibiotic resistance database (CARD) (Alcock et al., 2020). The CARD is a rigorously curated collection of characterized, peer-reviewed resistance determinants and associated antibiotics, organized by the antibiotic resistance ontology (ARO) and AMR gene detection models. *M. fortuitum* CT6 (CP011269.1) strain genome sequence of drug-resistant associated genes was obtained from the NCBI database which was used as a reference sequence. A BLAST search was done against the newly identified *M. fortuitum* complex member MF GZ001 strain using these genes as the query. To find virulence genes and virulence factors related genes, the protein sequences predicted by RAST were BLAST searched against the comprehensive online resource virulence factors database (VFDB) (Yang et al., 2007). We selected the genes that were orthologous to virulence genes with at least 60% identity and 60% sequence coverage in query and subject using our own Perl scripts. For the comparative study of virulence genes, the reference sequences were compared with *M. fortuitum* complex strains and 21 other mycobacterial species by using the same approaches to indicate the alterations in species-specific genes.

### Prophage and CRISPR predictions

For the prediction of prophage, the annotated sequence of the MF GZ001 genome was checked using PhiSpy software (version 4.1.16) (Akhter et al., 2012). Additionally, clustered regularly interspaced short palindromic repeats (CRISPRs) were detected in the genome by using MinCED (from the CRISPR recognition tool) (Bland et al., 2007; Grissa et al., 2007).

### Data availability

The MF GZ001 genome sequence data were deposited in the NCBI database under accession number CP107719. Raw data of WGS were deposited in the NCBI Sequence Read Archive (SRA) under the accession SRR22164027. All bacterial strains and analyses are illustrated in this manuscript and its **Supplementary materials**.

## Results

### Growth kinetics and morphology study

**Figure 1:**
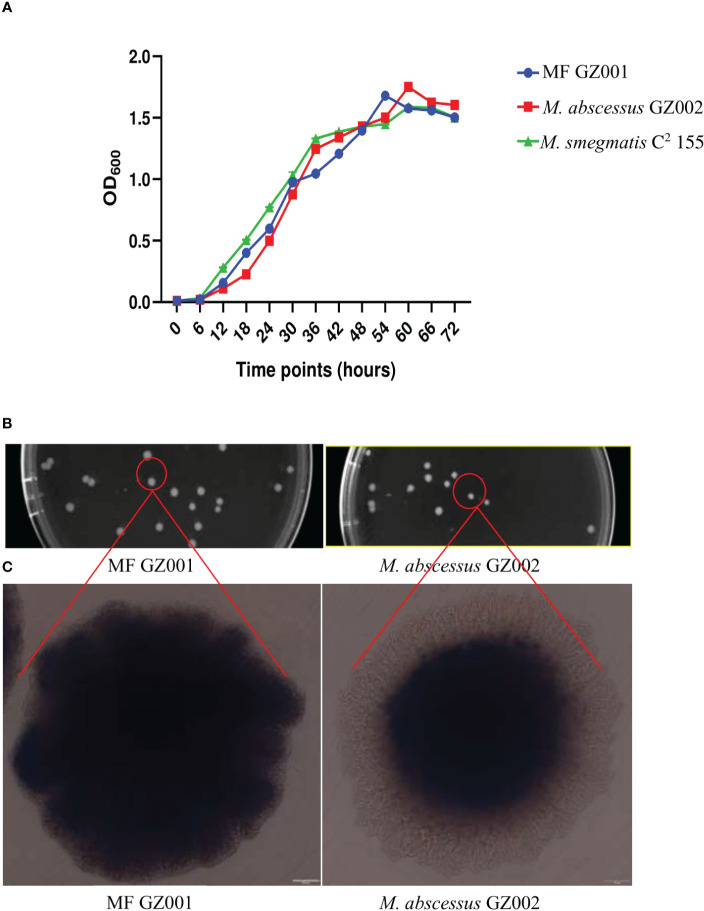
Growth kinetics and colony surface architecture of Mycobacterium species. **(A)** Growth kinetics detection of MF GZ001, *M. abscessus* GZ002, and *M. smegmatis* C^2^ 155. **(B)** Colony size and morphology determination. MF GZ001 and *M. abscessus* GZ002 were grown in Middlebrook 7H11 agar media supplemented with Tween 80 at 37°C. **(C)** Individual colony surface architecture was measured by OLYMPUS TH4-200.

To compare the growth kinetics of MF GZ001 and other NTM, growth curves were plotted for MF GZ001, *M. abscessus* GZ002, and *M. smegmatis* C^2^ 155. After culturing in Middlebrook 7H9, OD_600_ was determined at 6 hrs intervals to detect bacterial growth rates. Both MF GZ001 and *M. smegmatis* C^2^ 155 strains have shown faster growth than *M. abscessus* GZ002 strain in terms of their OD_600_ at various time intervals and same growth conditions (**Figure 1A**), though the differences were not statistically significant (*P >* 0.05). To better understand mycobacterial morphology, the MF GZ001 and *M. abscessus* GZ002 strains were grown on 7H11 Middlebrook agar plates and incubated for 7 days at 37°C. We have noticed a colony shape of MF GZ001 slightly larger than the *M. abscessus* GZ002 strain by measuring the diameter of a single colony (**Figure 1B**). OLYMPUS TH4-200 microscopic visualization of colony structure revealed that MF GZ001 has a wrinkled colony surface and rough morphotype, whereas *M. abscessus* GZ002 colonies are non-wrinkled and exist in a smooth morphotype (**Figure 1C**).

### DST

We determined the *in vitro* susceptibility of MF GZ001 to several drugs using the broth micro-dilution method. The MF GZ001 strain showed high resistance to sulfamethoxazole (> 128 µg/mL), clofazimine (> 128 µg/mL), levofloxacin (> 16 µg/mL), carbapenem (> 128 µg/mL), RIF (64 µg/mL), imipenem (64 µg/mL), and streptomycin (64 µg/mL). The susceptibility of MF GZ001 to other therapeutic agents was also determined (**Table S11**).

### General overview of MF GZ001 genome

**Figure 2:**
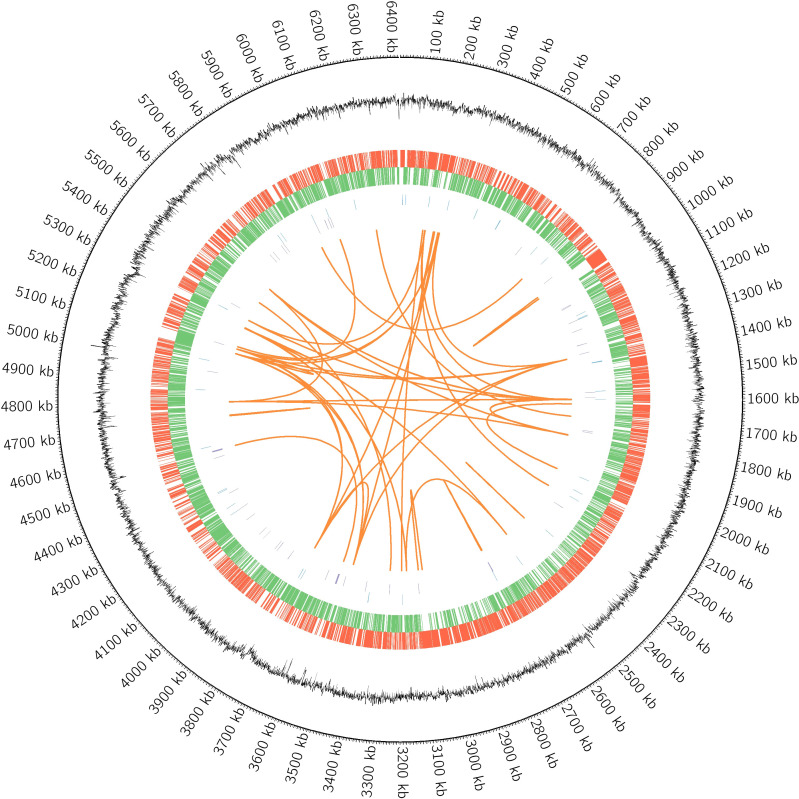
Circular representation of MF GZ001 genome displayed with Circos (version 0.69). The circular plot has seven levels. From outside to inside, the first is the information of genome position, the second is GC content information, the third is positive strand genes (marked in red color), the fourth is negative strand genes (marked in green color), the fifth is positive strand ncRNA data (marked in blue color), the sixth is negative strand ncRNA data (marked in purple color) and the seventh shows long repeats (>100 bp).

MF GZ001 genome was sequenced using SMRT sequencing technology and Illumina HiSeq sequencing platform at high sequencing depth. The mapping ratio of the genome was 98.79% which covered 100% reads of the genome (**Table S6**, **Figure S1**). For the paired-end data of sequencing, the total number of reads and bases counts were 19187140 and 2878071000 respectively. After trimming, the average length of sequence reads was 149.50 bp. The quality evaluation of Pacbio raw reads of sequences and bases obtained from MF GZ001 were 1368432 and 4062640505, respectively. The size of the MF GZ001 genome is 6413573 bp. The final assembly of the genome was circularized with a high G+C content of 66.18% (**Figure 2**, **Table 1**). It contained 6156 protein-coding genes, whereas protein-coding genes with enzymes were 1450. In addition, a total of 98 ncRNAs, 6 rRNAs, 55 tRNAs, and 37 other ncRNAs were identified (**Table 1**). Moreover, a prophage with approximate size of 39521 bp (**Table S7**) and three repeat numbers of clustered regularly interspaced palindromic repeats (CRISPR) were predicted in the genome (**Table S8**).

**Table 1:** General overview of MF GZ001 genome.

Features	Value	% of total
Size (bp)	6413573	100.00
G+C content (bp)	4244546	66.18
Coding region (bp)	5966586	93.03
Total genes	6254	100.00
Protein-coding genes	6156	98.43
Protein coding genes with enzymes	1450	23.19
RNA genes	98	1.57
Total number of ncRNAs	98	N/A
Number of rRNAs	6	N/A
Number of tRNAs	55	N/A
Number of other ncRNAs	37	N/A
Genes assigned to COGs	4315	69.00
COG clusters	1613	37.38
Genes with signal peptides	495	7.91
Genes with transmembrane helices	1428	22.8

N/A, Not Applicable.

### Phylogenetic construction analysis

The phylogenetic taxonomic position of *M. fortuitum* MF GZ001 within the mycobacterium genus was constructed based on FastTree and Mafft’s alignment of the target sequence of 33 mycobacterial species. Initially, the phylogenetic tree was constructed based on the worldwide known bacterial identification marker genes *16S rRNA* and *hsp65*. The *16S rRNA*-based phylogenetic tree indicated that MF GZ001 was the closest sub-species to *M. fortuitum* (**Figure S8A**). Among several mycobacterium species, the other closest members were *M. phocaicum, M. mucogenicum, M. septicum, and M. bacteremicum*, which belongs to *M. fortuitum* complex. In addition*, hsp65-*based phylogenetic analysis showed that MF GZ001 is closely related to *M. fortuitum* W4 (**Figure S8B**), which suggests that MF GZ001 is a member of the *M. fortuitum* complex. Later on, we reconstructed a phylogenetic tree based on multiple gene approaches (*16S rRNA*, *hsp65*, *rpoB*) to further confirm the placement of MF GZ001. The multiple gene-based reconstructed phylogenetic results were most related to *M. fortuitum* CT6 (**Figure S8C**) and distantly related to SGM. Finally, we subjected MF GZ001 to ANI analysis using the whole genome sequences to clarify the inconsistency from the single gene-based phylogenetic predictions. The ANI values between MF GZ001 and *M. fortuitum* CT6, *M. alvei* CIP103464, *M. septicum* DSM44393, and *M. mageritense* JR2009 strains were 98.77%, 86.26%, 86.53%, and 80.38% respectively. This study revealed that the MF GZ001 strain shares the most genetic relatedness with *M. fortuitum* CT6.

### Functional annotation study

The functional pathways of annotated genes were interpreted using protein-coding genes of the MF GZ001 strain in the KEGG database. A total of 2411 genes were annotated to six-factor types and 40 KEGG functional pathways along with 558 for amino acid metabolism (**Figure S2** **;** **File.Xls.S1** **)**. In addition, there were 23 genes which are possibly related to different pathways in cancer, for instance central carbon metabolism in cancer, proteoglycans in cancer, K005215 prostate cancer, and K05219 bladder cancer. These genes are not only related to cancer-associated pathways but also other pathways like lipid metabolism, pyridine metabolism, and drug metabolism. Moreover, 1 aquaporin z, 113 uncharacterized proteins, drug resistance genes, and human infectious diseases-related genes (**File.Xls.S1**). COG functional annotation of the MF GZ001 genome was studied by the COG database. A total of 4315 common COGs have been found in the MF GZ001 genome. For the MF GZ001 strain, the genes were functionally categorized into 21 different groups (**Figure S3**; **File.Xl.S2**). The general and unknown functions of functionally annotated MF GZ001 genome sequence have been predicted by R and S categories respectively. Notably, there were 361 genes found as unknown function categories and no homologs were identified in the COG database. The majority of the remaining functional annotation categories were represented by “transcription (K category; 530), lipid transport and metabolism (I category; 486), secondary metabolites biosynthesis, transport, and catabolism (Q category; 434), energy production and conversion (C category; 385), amino acid transport and metabolism (E; category; 383) (**File.Xl.S2**). In addition, the non-redundant homologous species distribution map was created for comprehensive information using the NCBI database. Among different mycobacterial species, the highest (64.83%) similarity was found with *M. fortuitum* (**Figure S4**, **File Xl.S3**). However, other analyses based on blast to GO, CAZy, Pfam, and Swiss_Prot database gene annotation result statistics table have been shown in **supplementary materials** (**Figures S5, S6**, **Table S9**, **File. Xl.S4, S5**).

### Comparative genomic analysis

To investigate the genomic evolution of MF GZ001 strain, based on phylogenetic construction analysis and ANI analysis results, we compared the MF GZ001 strain with the more closely related species *M. fortuitum* CT6 as well as other RGM and SGM mycobacterium species. The evolutionary results emphasized that the MF GZ001 strain has a close relationship with *M. fortuitum* subsp*. fortuitum*. The reannotation results revealed that MF GZ001 strain genome size (6413573 bp) and CDS sequences were higher than *M. fortuitum* CT6 (6254616 bp) (**Table 2**). Interestingly, the MF GZ001 has the highest number of conserved unknown functional genes (1157) compared to *M. fortuitum* CT6, *M. abscessus* GZ002, *M. smegmatis* C^2^155, and *M. tuberculosis* H37Rv (**Table 2**). Additionally, the MF GZ001 genome contains more unique genes (333) than the reference genome *M. fortuitum* CT6 (264) and has more gene density (base pairs per gene) compared to other mycobacterial genomes (**Table 2**). Moreover, the analysis endorsed that the MF GZ001 strain is substantially diversified compared to the *M. fortuitum* CT6 strain.

**Table 2:** Chromosomal features of MF GZ001 compared to other RGM and SGM.

Features	MF GZ001	*M. fortuitum* CT6	*M. abscessus* GZ002	*M. smegmatis* C^2^ 155	*M. tuberculosis* H37Rv
Chromosome size (base pairs)	6413573	6254616	6993871	5067231	4411532
G+C (%)	66.18	66.22	64.13	67.39	65.61
Gene density (base pairs per gene)	1044.88	1039.11	1062.62	1033.41	1001.4
Average CDS length	969.23	969.78	949.29	973.43	1013.39
Protein-coding sequences (CDS)	6156	5892	4927	6531	3906
Conserved with assigned function	4999	4944	4126	5818	2851
Conserved with unknown function	1157	948	801	713	1055
Unique	333	264	1303	1820	1025
Pseudogenes	0	96	32	124	0
rRNA	6	6	3	9	3
tRNA	55	52	47	47	45

The advantage of high accuracy in obtaining *de novo* sequencing is to identify both major and minor variations in the genome that can reveal the origin of strains or have effects on the gene function. For instance, we have detected 74168 single-nucleotide variations (SNVs) and 2001 INDEL mutations in the MF GZ001 strains compared to the reference genome (**File.Xl.S6**). The sequencing data produced 50914 synonymous, 17563 non-synonymous SNVs, and 2001 INDEL mutation (**Table 3**). Interestingly, there were 174 intergenic mutations belonging to the two genes (*1_3425*, *1_3426*), and also obtained stop loss (33) and stop gain (110) variants in the MF GZ001 strain (**File.Xl.S6** and **Table 3**). To know the divergence of other mycobacterium species, we investigated the SNVs data of MF GZ001 not only comparing it with closely related species but also with distantly related species. Additionally, the highest number of SNVs was obtained from MF GZ001 compared to *M. smegmatis* C^2^ 155. Specific positions of SNVs/INDEL in genes and mapping distribution of SNVs have been illustrated in **Supplementary materials** (**File.Xl.S6** and **Figure S7**).

**Table 3:** Single-nucleotide variations (SNVs) identified in MF GZ001 strain compared with the reference strains.

Identified strain	Reference strains	Synonymous	Non-synonymous	INDEL	Stop loss	stop gain
MF GZ001	*M. fortuitum* CT6	50914	17563	2001	33	110
	*M. abscessus* GZ002	25843	12261	1469	11	151
	*M. smegmatis* C^2^ 155	190593	83927	10764	203	875
	*M. tuberculosis* H37Rv	33109	15673	1887	16	181

**Figure 3:**
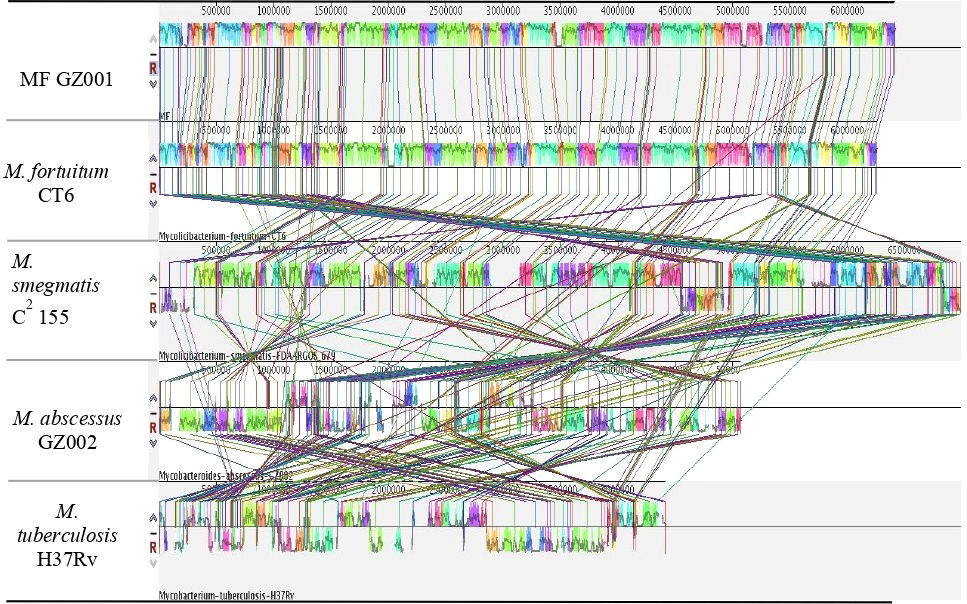
Diagram depicting genomic comparisons obtained using Mauve software. The alignment display is organized into one horizontal “panel” per input genome sequence. Each genome’s panel contains the name of the genome sequence, a scale showing the sequence coordinates for that genome, and a single black horizontal center line. Colored block outlines appear above and possibly below the center line. Each of these block outlines surrounds a region of the genome sequence that is aligned to part of another genome and is presumably homologous and internally free from genomic rearrangement. Regions outside blocks lack detectable homology among the input genomes. Inside each block, Mauve draws a similar profile of the genome sequence. The height of the similarity profile corresponds to the average level of conservation in that region of the genome sequence. Areas that are completely white were not aligned and probably contain sequence elements specific to a particular genome. The height of the similarity profile is calculated to be inversely proportional to the average alignment column entropy over a region of the alignment.

**Figure 4:**
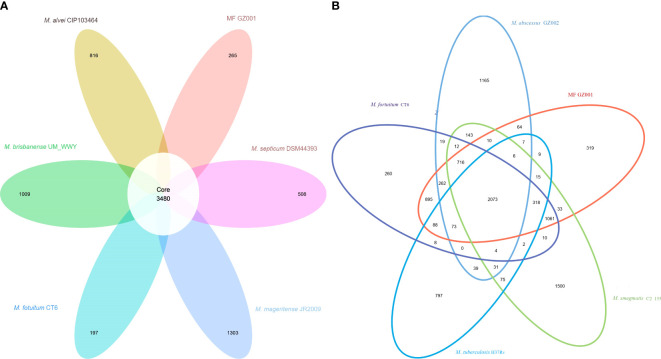
Comparative genomes representation. **(A)** Pan-genome of *M. fortuitum* complex. The white circle in the middle of the flower displayed core gene clusters, and the petals represent the unique number of clusters of each Mycobacterium species. **(B)** MF GZ001 and other mycobacterial strains genome-based Venn diagrams. The circles of different colors in the Venn diagram represent different species, and the numbers in the figure represent the numbers of gene families unique or common to each strain. In the petal diagram, each petal represents a specie. The numbers on the petals represent the number of gene families unique to the species, and the white circle in the middle represents the number of gene families shared by all strains.

To study the genome modification, the collinearity analysis of the MF GZ001 strain compared to the *M. fortuitum* CT6, *M. abscessus* GZ002, *M. smegmatis* C^2^ 155, and *M. tuberculosis* H37Rv was performed. The collinearity outcomes predicted from the MF GZ001 genome sequence was more aligned with the *M. fortuitum* CT6 strain, exhibiting no significant genomic modifications (**Figure 3**). The highest matching collinearity results are consistent with our previous analysis. Using the blast core ratio algorithm, we have investigated the MF GZ001*, M. alvei* CIP103464, *M. fortuitum* CT6*, M. brisbanense* UM_WWY, *M. septicum* DSM44393, and *M. mageritense* JR2009 genome to determine the account number of core gene clusters, pan-gene clusters, specific gene clusters, unique, and other gene clusters. There were 11503 pan-gene clusters with 38843 genes, 3480 core gene clusters (21621 genes), and 4098 specific gene clusters (4359 genes) (**Figure 4A**). The comparative analysis of the pan-genome of the *M. fortuitum* complex detected lower and nearly similar unique gene clusters of MF GZ001 (265) with *M. fortuitum* CT6 (197) than other *M. fortuitum* complex members (**Figure 4A**). This hypothesis revealed that MF GZ001 species had a significant amount of genomic variation, which was to be noted in the pan-open genome’s structure and close to the *M. fortuitum* complex. Moreover, the genome sequence of MF GZ001 was compared with *M. fortuitum* CT6, *M. abscessus* GZ002, *M. smegmatis* C^2^ 155, and *M. tuberulosis* H37Rv to estimate the number of gene clusters that are shared between each strain. Ven diagram study predicted that the core and specific gene clusters were 2073 and 4041, respectively (**Figure 4B**). MF GZ001 shared the highest number of gene clusters (2352) with *M. fortuitum* CT6 than other closely related species *M. abscessus* GZ002 (1138), *M. smegmatis* C^2^ 155 (2126), and distantly related *M. tuberculosis* H37Rv (526) species. The reference strain *M. forturuim* CT6 obtained 260 unique gene clusters, whereas the MF GZ001 strain obtained 319 unique gene clusters which are related to the metabolic, molecular, and protein regulatory function of bacteria (**Figure 4B**).

### Drug resistance profile and variants found in the MF GZ001 strain

**Figure 5:**
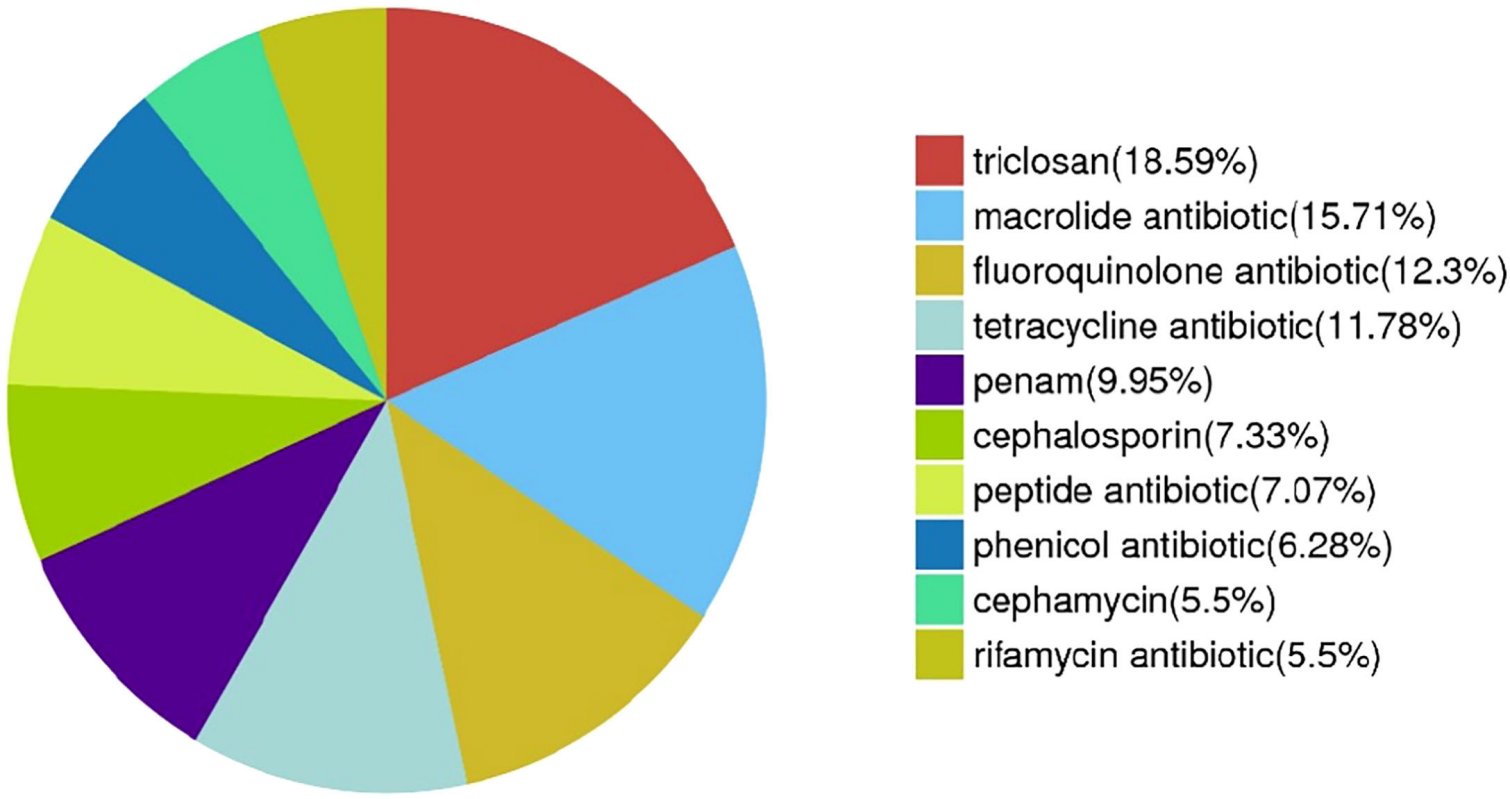
Drug resistance-related genes distribution in MF GZ001 strain.

From a comprehensive drug resistance analysis, the MF GZ001 strain encodes multiple drug-resistant related genes against important drugs, including RIF, macrolides, fluoroquinolones, tetracyclines, triclosan, penem, peptide antibiotics, and cephamycin. The analysis reveals that the highest (18.59%) drug-resistant related genes were associated with triclosan (71), 15.71% macrolides (60), 12.3% fluoroquinolones (47), tetracyclines (45), 5.5% RIF (21) and 7.33% cephalosporin (28) (**Figure 5**; **Table S10**). Moreover, the prevalence of the underlying SNP mutations was found in the most comprehensive drug resistance-related genes, for example, *rpoB, katG, AAC(2’)-Ib, gyrA, gyrB, embB, pncA, blaF, thyA, embC, embR* and *iniA.* The most prevalent and more than 60% identity resistance-related genes have been shown in **supplementary materials** (**Table S13**). The SNP investigations showed some mutations that are not commonly observed in RGM species. For instance, the mutations in *iniA, iniC, pncA*, and *ribD* are conferred resistance to isoniazid, ethambutol, pyrazinamide, and para-aminosalicylic acid in *M. tuberculosis* (**Table S13**). Additionally, we have detected a mutation in the *AAC (2’)-Ib* gene that causes streptomycin resistance and is more frequent in the *M. fortuitum* complex.

### Comparative study of virulence genes

The virulence genes analysis reveals that the MF GZ001 genome included 167 putative virulence genes that are shared by other SGM and RGM pathogenic mycobacterium species (**File.Xl.S8**). We have studied the predicted virulence gene clusters across the MF GZ001 and 21 other mycobacterial strains (**Table S14**) based on the existence and non-existence of specific genes which indicated the relationship status between MF GZ001 and *M. fortuitum* complex. The identified 167 putative virulence genes in the mycobacterium species are responsible for inducing nitrate reductase activity, mycobacterial cell envelope, inhibition of apoptosis, lipid and protein metabolism, gene regulation, and resistance of anti-mycobacterial agents. For example, the MF GZ001 genome contains *nark2* non-redundant virulence genes which encode the nitrite transporter proteins related to exhorting nitrate reductase activity in response to reducing oxygen level by regulating its transcriptional regulation level whereas *narH*, *narG*, and *narI* (**Table S14**) genes encoded the proteins responsible for nitrate respiration in the absence of oxygen in the *M. tuberculosis* and *M. fortuitum* complex (Weber et al., 2000; Giffin et al., 2012). The nitrate reductase virulence factor lack in other mycobacterial species like *M. abscessus*, *M. leprae, M. marinum*, and *M. ulcerans*. Importantly, we also reported the virulence genes in MF GZ001 for instance *fbpA*, *fbpB*, *fbpC*, *hbhA*, and *mce*, encoded proteins belonging to antigen 85 complex locus (**File.Xl.S8**) essential for the synthesis of the bacterial cell wall (Armitige et al., 2000). These genes encoded proteins function is mycolyltransferase activity, which is essential for the development of the cell wall and the survival of mycobacteria (Mandato and Chai, 2018). We have identified five sigma factors (transcription initiation factors) that are linked to virulence in *M. tuberculosis* H37Rv (*SigAP/rpoV*, *sigE*, *sigF*, *sigH* and *sigL*), as well as the mammalian cell entry *mce* operons (**Table S14**) that are extensively distributed across mycobacteria (Arruda et al., 1993; Wee et al., 2016). The *mce* operon has been demonstrated to be crucial for mycobacterial invasion and persistence in host macrophages and non-phagocytic mammalian cells, with *mce4* being associated with cholesterol catabolism (Zhang and Xie, 2011; Griffin et al., 2011). The MF GZ001 genome has seven operons *mce1*, *mce3*, *mce4*, *mce5*, *mce6*, *mce7*, and *mce8*, and the absence of *mce2* and *mce9* operons. MF GZ001 contained *mce1* operon homolog to *M. tuberculosis* but has not been detected in most therapeutic-resistant pathogen *M. abscessus*.

The Type VII secretion machinery is essential for mycobacterial pathophysiology and virulence (Groschel et al., 2016). Mycobacterium species include ESX 1 to 5 virulence factors and ESX-2 and ESX-5 factors are noticed in SGM. The ESX-5 is only present in SGM that could appear differentiated between RGM and SGM mycobacteria (Beckham et al., 2017). MF GZ001 genome contained ESX-1 (*espR, mycP1, eccD1, espI, esxA, eccA1, eccB1, eccCa1, eccCb1, PPE68, esxB*) virulence factor, without *espJ*, and *espK* genes that are related to SGM (**File.Xl.S8**). This result is consistent with *M. fortuitum*. The ESX-1 virulence factor is considered a significant virulence determinant (Madacki et al., 2021) that is associated with the inhibition of T-cell responses, inducing the differentiation of macrophages into foam cells (MacGurn and Cox, 2007; Samten et al., 2009) as well as aids in the escape of mycobacteria from phagosomes by ESAT-6 mediated perforation of vacuolar membrane (Smith et al., 2008). Moreover, we have reported that ESX-3 included *esxG, esxH, espG3, eccD3, mycP3, eccE3, eccA3, eccB3*, and *eccC3* virulence-related genes in the MF GZ001 genome. The ESX-3 factor is present in all mycobacteria and specifically essential for *in vitro* growth in *M. tuberculosis* (Sassetti et al., 2003). The ESX-3 factor is involved in iron homeostasis and uptake through the mycobactin pathway (Newton-Foot et al., 2016).

We found novel putative virulence genes homologs to *Rv1837c*, *groEL2*, *Rv0926c*, and *Rv0204c* associated with malate synthesis, dendritic cell responses, uncharacterized hypothetical protein, and transmembrane-related protein respectively that may only present in MF GZ001 strain and *M. tuberculosis* H37Rv (**File.Xl.S8**). Additionally, we have also identified unknown function virulence factors in the MF GZ001 strain which are related to RGM and SGM.

Furthermore, the MF GZ001 strain includes genes homologs to *katG*, *sodA*, and *sodC* that encode enzymes such as catalase-peroxidase and superoxide dismutase A which are responsible for oxidative stress tolerance (Forrellad et al., 2013). These genes may be essential for preventing the oxidative burst that occurs during macrophage survival. We have identified *nuoG* which is considered a key virulence gene of *M. tuberculosis* that encodes NADH dehydrogenase type I complex protein in the mycobacterial membrane. Similarly, some other important virulence genes are also identified for instance *secA2*, and *ptpA* (Cossu et al., 2012). These genes have been implicated as an antiapoptotic factor for macrophages (Velmurugan et al., 2007). Consequently, these genes enable the mycobacteria to avoid the host cells’ built-in mechanism for cell death.

## Discussion

Immunocompromised patients are particularly vulnerable to NTM infections, and the incidence of NTM infections has significantly increased globally in recent decades (Dohal et al., 2021). In this investigation, we have provided the whole genome sequencing and comparative genome analysis of a clinical strain of NTM species, MF GZ001. In bacterial growth kinetic analysis, there was no statistically significant growth difference compared with other RGM, indicating that MF GZ001 is an RGM NTM species. The MF GZ001 has a wrinkled colony surface and rough morphotype which are consistent with a previous study (Gharbi et al., 2021). The rough morphotype *M. fortuitum* and *M. abscessus* comparatively possess more pathogenic potential (Brambilla et al., 2016; Gharbi et al., 2021). Recently, Dedrick et al., predicted the prophage length from 39.1 kb bp to 80.7 kb in clinical isolates, with an average size of ∼55.3 kbp (Dedrick et al., 2021), which was closely related to prophage (39.521 kb) from our clinical strain MF GZ001 and distantly related to prophage (<11.0 kb) from *M. smegmatis* (Jordan et al., 2014; Sewell, 2017). The average prophage size indicates that the *M. fortuitum* MF GZ001 pathogenic profile may be the same as *M. abscessus*. Additionally, the DST revealed MF GZ001 strain was highly resistant to most of the therapeutic agents which also indicates that MF GZ001 strain is more virulent. Based on a single marker gene and multiple gene approaches, the phylogenetic study and ANI revealed that MF GZ001 is closely related to the *M. fortuitum* CT6. The functional reannotation analysis revealed the new mycobacterium species MF GZ001 (6254616 bp) genome size is larger than that of *M. fortuitum* CT6 (6254616 bp). Interestingly, MF GZ001 contains the highest number of conserved unknown functional genes compared to *M. fortuitum* complex members, and other RGM and SGM mycobacterium species. Moreover, MF GZ001 included more unique genes (333) than the reference genome *M. fortuitum* CT6 (264). The larger genome size of MF GZ001 may reveal the diverse genomic structure of *M. fortuitum* complex. This diverse structure was observed may be due to horizontal gene transfer, CRISPR elements, and the prophage found in this species which may have an important impact on the fitness and pathogenicity of their bacterial hosts (Cote et al., 2022). According to previous studies of prophage in NTM clinical strains, prophage was found more likely in RGM than SGM (Dedrick et al., 2021; Senhaji-Kacha et al., 2021). The clinical NTM prophages may have more abundant virulence genes than the prophages from the environmental mycobacteria (Glickman et al., 2020). Based on an overview of the MF GZ001 genome, the prophage analysis predicted that it contains a (39.521 kb) prophage region. The prophage sequence is flanked by 13 bp phage attachment sites, *attL*, and *attR* (**Table S7**). Though it has been studied that clinical NTM exhibits various levels of virulence, but it is not thoroughly verified yet that whether the prophage elements containing virulence genes have any impact on treatment outcomes (Gonzalez-Perez et al., 2013). The existence of virulence genes does not imply that they are being actively expressed, and the presence of a prophage in a genome does not prove that the virus is excisable or functional (Glickman et al., 2020). According to the literature contradictory review, it would be beneficial to conduct further research to characterize the prophage of the *M. fortuitum* complex. Mycobacterium species can also transfer DNA intrinsically and incorporate DNA from foreign sources between species (Rabello et al., 2012; Wee et al., 2016). In addition to comparative analysis based on genomes such as higher level of colinearity, a Venn diagram, and a pan-genome study showed a close relationship between MF GZ001 and *M. fortuitum* CT6. Although the genome variation was also observed, more characteristics were shared with *M. fortuitum* complex members than other mycobacterium species. This investigation uncovered a close link between MF GZ001 and *M. fortuitum* complex which provides insight into the deep evolution of pathogenesis in RGM species.

To our knowledge, this is the first report of SNV/INDEL mutation based on *de novo* sequencing methods in *M. fortuitum* complex members. In this report, we have detected SNV/INDEL mutations compared to the reference strains and other mycobacterium species. We have identified 74168 SNVs and 2001 INDEL mutations in the MF GZ001 strains compared to the reference genome. Moreover, we have identified stop loss (33) and stop gain (110) variants in the MF GZ001 strain. Most putative resistance mutations were observed in nonessential areas where hundreds of loss-of-function mutations might cause resistance (Miotto et al., 2017). Several variants conferred in a loss of function due to INDEL or stop codons (Richard et al., 2019). Importantly, we have found 174 intragenic mutations in two genes (*1_3425, 1_3426*) that are of unknown function. This study further emphasizes investigating the role of genes and the therapeutic resistance mechanisms in the RGM *M. fortuitum* complex.

Furthermore, the MF GZ001 drug resistance profile was studied *via in vitro* DST and prediction of drug resistance genes and variants with *in silico* analysis. *M. fortuitum* infected cases resistant to clarithromycin and fluoroquinolones have also been reported previously (Kurokawa et al., 2020)*. In vitro* testing of MF GZ001 showed high resistance to macrolides, clofazimine, sulfamethoxazole, levofloxacin, streptomycin, and RIF. No frequent studies have been conducted to investigate the *M. fortuitum* complex. In this first report, drug resistance gene prediction through *in silico* approaches showed 18.59% genes associated with resistance to triclosan, 15.71% to macrolides, and 12.3% to fluoroquinolones. We have found SNPs in drug resistance-related genes such as *rpoB, arr, AAC(2’)-Ib, katG, gyrA, gyrB, embB, blaF, thyA, embC* and *embR* that are previously reported in *M. fortuitum* and other mycobacterium species (Nash et al., 2005; Ramon-Garcia et al., 2006). For instance, among them recently reported, the ribosylation ADP-ribosyltransferase *arr* gene in *M. fortuitum* 7G was linked to the high level of RIF resistance which is consistent with our findings (Morgado et al., 2022a). A mutation in the *rpoB* gene’s particular region known as the rifampin-resistance-determining region (RRDR) leads to the emergence of RIF resistance in NTM and *M. tuberculosis* (Saxena et al., 2021). Additionally, by expressing the *arr* gene, *M. abscessus* and *M. smegmatis* intrinsically reduced the function of RIF (Brown-Elliott et al., 2012). The *erm* gene may responsible for the intrinsic resistance to macrolides in *M. fortuitum* strains. Erm is a member of a large family of proteins that are encoded by a variety of alleles, some of which (*erm*37-41) are specifically linked to Mycobacteriaceae species (Nash et al., 2005). Furthermore, similar to our study the mutations in *gyrA* have also been reported to be responsible for quinolone resistance in *M. fortuitum* (Kamada et al., 2021).

Importantly, the SNPs found in genes of MF GZ001, for example, *iniA, iniC, pncA*, and *ribD* conferring resistance to isoniazid, ethambutol, pyrazinamide, para-aminosalicylic acid, and isoniazid resistance respectively, are not commonly observed in RGM. We have reported a mutation in a distinctive gene *aph (3”)-Ic* that is related to streptomycin resistance which is consistent with other studies (Morgado et al., 2022a). The *aph (3”)-Ic* distinctive gene was first reported in environmental *M. fortuitum* and later in clinical isolates as well (Ramon-Garcia et al., 2006; Morgado et al., 2022b). We anticipate that further investigation of the novel SNPs in *iniA, iniC, ribD, pncA*, and *aph (3”)-Ic* detected in this study are necessary to explore their role in the development of resistance. Particularly, these well-known drug-resistant genes are found in the MF GZ001 strain, which will be useful for further study on strain identification and characterizations enriching the genetic data sources for pathogenic diseases.

The genome of MF GZ001 comprises a variety of putative virulence genes that may have enhanced its ability for intracellular replication and persistence. *M. fortuitum* exhibits robust pathogenicity linked to various virulence genes, for instance, *mce*, *nar*, sigma, and antigen 85 complex clusters. These genes could encode potential virulence factors associated with mycobacterial cell envelope, inhibition of apoptosis, lipid and protein metabolism, gene regulation as well as nitrate reductase activity. The *mce* operons distributed across mycobacterium species are responsible for mycobacterial invasion and persistence in host macrophages and non-phagocytic mammalian cells, with *mce4* being associated with cholesterol catabolism (Zhang and Xie, 2011). *M. fortuitum* contains the *nuoG* gene, which encodes NADH dehydrogenase type I complex protein in the mycobacterial membrane which is considered as an emergent virulence factor in *M. tuberculosis* (Velmurugan et al., 2007; Cossu et al., 2012). MF GZ001 genome contains an important *trpD* gene which is also confirmed by the previous study. Among RGM, a gene copy of *trpD* was only detected in *M. abscessus* and *M. fortuitum* complex members (Gharbi et al., 2021). It encodes an anthranilate phosphoribosyltransferase associated with tryptophan biosynthesis which has contributed a significant role during infection of patients for SGM (Zhang et al., 2013). Particularly, the MF GZ001 genome has *katG*, *sodA* gene for oxidative stress resistance which may be crucial for preventing the oxidative burst that occurs during macrophage survival. These genes have been shown by Wee et al., in *M. brisbanense* which is a member of the *M. fortuitum* complex (Wee et al., 2016). Furthermore, in recent years it has been identified that the genes responsible for the biosynthesis of the many unique lipids are found in the mycobacterial cell wall. For instance, MF GZ001 contains the acyl-coenzyme A (CoA) synthase gene (*fadD28*) which is associated with the esterification of the acid to phthiocerol to produce dimycocerosyl phthiocerol which is involved in virulence (Fitzmaurice and Kolattukudy, 1997; Sirakova et al., 2002; Brodin et al., 2010).

Furthermore, we reported novel putative virulence genes that are homologous to *Rv1837c, groEL2, Rv0926c*, and *Rv0204c*, and are related to malate synthesis, dendritic cell responses, an unidentified hypothetical protein, and a transmembrane-related protein, respectively. These genes may only be found in the MF GZ001 strain and *M. tuberculosis* H37Rv. Recently, Luo et al., reported that the secretion proteins locus ESX-1, ESX-3, and ESX-5 are crucial for virulence in *M. tuberculosis* (Luo et al., 2022) and were found in the MF GZ001 strain which is consistent with the previous report (Gharbi et al., 2021; Morgado et al., 2022b). Therefore, the MF GZ001 genome sequence will provide insights into *M. fortuitum* complex potential pathogenesis. The existence of the potential virulence determinants still requires experimental evidence to detect whether the MF GZ001 strain is associated with crucial human diseases or not.

In summary, we have sequenced and analyzed the genome of the MF GZ001 strain that might be a new member of the *M. fortuitum* complex. Our comparative analysis indicated that it is a new member of the *M. fortuitum* complex and a human-pathogenic species. The findings of this study will establish a foundation for further investigations of the pathogenic mechanism of *M. fortuitum* as well as its diagnosis and treatment, especially in southern China.

## Data availability statement

The original datasets generated in the study are publicly available. The MFGZ001 WGS and SRA data were deposited in the NCBI database under accession numbers CP107719 and SRR22164027.

## Author contributions

All authors contributed to the article and approved the submitted version.

## References

[B1] Aitken M. L. Limaye A. Pottinger P. Whimbey E. Goss C. H. Tonelli M. R.. (2012). Respiratory outbreak of *Mycobacterium abscessus* subspecies *massiliense* in a lung transplant and cystic fibrosis center. Am. J. Respir. Crit. Care Med. 185 (2), 231–232. doi: 10.1164/ajrccm.185.2.231 22246710

[B2] Akhter S. Aziz R. K. Edwards R. A. (2012). PhiSpy: a novel algorithm for finding prophages in bacterial genomes that combines similarity-and composition-based strategies. Nucleic Acids Res. 40 (16), e126–e126. doi: 10.1093/nar/gks406 22584627 PMC3439882

[B3] Alcock B. P. Raphenya A. R. Lau T. T. Tsang K. K. Bouchard M. Edalatmand A.. (2020). CARD 2020: antibiotic resistome surveillance with the comprehensive antibiotic resistance database. Nucleic Acids Res. 48 (D1), D517–D525. doi: 10.1093/nar/gkz935 31665441 PMC7145624

[B4] Armitige L. Y. Jagannath C. Wanger A. R. Norris S. J. (2000). Disruption of the genes encoding antigen 85A and antigen 85B of *Mycobacterium tuberculosis* H37Rv: effect on growth in culture and in macrophages. Infect. Immun. 68 (2), 767–778. doi: 10.1128/IAI.68.2.767-778.2000 10639445 PMC97204

[B5] Arruda S. Bomfim G. Knights R. Huima-Byron T. Riley L. W. (1993). Cloning of an *M. tuberculosis* DNA fragment associated with entry and survival inside cells. Science 261 (5127), 1454–1457. doi: 10.1126/science.8367727 8367727

[B6] Austin C. M. Tan M. H. Harrisson K. A. Lee Y. P. Croft L. J. Sunnucks P.. (2017). *De novo* genome assembly and annotation of australia's largest freshwater fish, the Murray cod (*Maccullochella peelii*), from illumina and nanopore sequencing read. GigaScience 6 (8), gix063. doi: 10.1093/gigascience/gix063 PMC5597895 28873963

[B7] Beckham K. S. Ciccarelli L. Bunduc C. M. Mertens H. D. Ummels R. Lugmayr W.. (2017). Structure of the mycobacterial ESX-5 type VII secretion system membrane complex by single-particle analysis. Nat. Microbiol. 2 (6), 1–7. doi: 10.1038/nmicrobiol.2017.47 28394313

[B8] Bland C. Ramsey T. L. Sabree F. Lowe M. Brown K. Kyrpides N. C.. (2007). CRISPR recognition tool (CRT): a tool for automatic detection of clustered regularly interspaced palindromic repeats. BMC Bioinf. 8 (1), 1–8. doi: 10.1186/1471-2105-8-209 PMC1924867 17577412

[B9] Brambilla C. Llorens-Fons M. Julian E. Noguera-Ortega E. Tomas-Martinez C. Perez-Trujillo M.. (2016). Mycobacteria clumping increase their capacity to damage macrophages. Front. Microbiol. 7. doi: 10.3389/fmicb.2016.01562 PMC5047892 27757105

[B10] Brodin P. Poquet Y. Levillain F. Peguillet I. Larrouy-Maumus G. Gilleron M.. (2010). High content phenotypic cell-based visual screen identifies *Mycobacterium tuberculosis* acyltrehalose-containing glycolipids involved in phagosome remodeling. PLos Pathog. 6 (9), e1001100. doi: 10.1371/journal.ppat 20844580 PMC2936551

[B11] Brown-Elliott B. A. Mann L. B. Hail D. Whitney C. Wallace J. R.J. (2012). Antimicrobial susceptibility of nontuberculous mycobacteria from eye infections. Cornea 31 (8), 900–906. doi: 10.1097/ICO.0b013e31823f8bb9 22362004

[B12] Brown-Elliott B. A. Philley J. V. (2017). Rapidly growing mycobacteria. Microbiol. Spectr. 5 (1), 703–723. doi: 10.1128/microbiolspec.TNMI7-0027-2016 PMC11687460 28084211

[B13] Brown-Elliott B. A. Wallace J. R.J. (2002). Clinical and taxonomic status of pathogenic nonpigmented or late-pigmenting rapidly growing mycobacteria. Clinc. Microbiol. Rev. 15 (4), 716–746. doi: 10.1128/CMR.15.4.716-746.2002 PMC126856 12364376

[B14] Bryant J. M. Grogono D. M. Greaves D. Foweraker J. Roddick I. Inns T.. (2013). Whole-genome sequencing to identify transmission of *Mycobacterium abscessus* between patients with cystic fibrosis: a retrospective cohort study. Lancet. 381 (9877), 1551–1560. doi: 10.1016/S0140-6736(13)60632-7 23541540 PMC3664974

[B15] Chan E. D. Iseman M. D. (2013). Underlying host risk factors for nontuberculous mycobacterial lung disease. Semin. Respir. Crit. Care Med. 34 (01), 110–123. doi: 10.1055/s-0033-1333573 23460011

[B16] Chhotaray C. Wang S. Tan Y. Ali A. Shehroz M. Zhang T.. (2020). Comparative analysis of whole-genome and methylome profiles of a smooth and a rough *Mycobacterium abscessus* clinical strain. G3 (Bethesda) 10 (1), 13–22. doi: 10.1534/g3.119.400737 31719113 PMC6945021

[B17] Cossu A. Sechi L. A. Zanetti S. Rosu V. (2012). Gene expression profiling of *Mycobacterium avium* subsp. *paratuberculosis* in simulated multi-stress conditions and within THP-1 cells reveals a new kind of interactive intramacrophage behaviour. BMC Microbiol. 12 (1), 1–17. doi: 10.1186/1471-2180-12-87 22646160 PMC3416667

[B18] Costa K. C. Bergkessel M. Saunders S. Korlach J. Newman D. K. (2015). Enzymatic degradation of phenazines can generate energy and protect sensitive organisms from toxicity. mBio. 6 (6), e01520–e01515. doi: 10.1128/mBio.01520-15 26507234 PMC4626857

[B19] Cote J. Welch C. Kimble M. Archambault D. Ross J. C. Molloy S. D.. (2022). Characterization of the cluster MabR prophages of *Mycobacterium abscessus* and. Mycobacterium chelonae. G3 (Bethesda) 12 (9), jkac188. doi: 10.1093/g3journal/jkac188 35894699 PMC9434293

[B20] Daley C. L. Griffith D. E. (2002). Pulmonary disease caused by rapidly growing mycobacteria. Clin. Chest. Med. 23 (3), 623–632. doi: 10.1016/s0272-5231(02)00021-7 12370998

[B21] Dedrick R. M. Aull H. G. Jacobs-Sera D. Garlena R. A. Russell D. A. Hatfull G. F.. (2021). The prophage and plasmid mobilome as a likely driver of *Mycobacterium abscessus* diversity. mBio. 12 (2), e03441–e03420. doi: 10.1128/mBio.03441-20 33785627 PMC8092301

[B22] De Groote M. A. Huitt G. (2006). Infections due to rapidly growing mycobacteria. Clinc. Infect. Dis. 42 (12), 1756–1763. doi: 10.1086/504381 16705584

[B23] Delcher A. L. Bratke K. A. Powers E. C. Salzberg S. L. (2007). Identifying bacterial genes and endosymbiont DNA with glimmer. Bioinformatics 23 (6), 673–679. doi: 10.1093/bioinformatics/btm009 17237039 PMC2387122

[B24] Diaz M. A. A. Huff T. N. Libertin C. R. (2019). Nontuberculous mycobacterial infections of the lower extremities: A 15-year experience. J. Clin. Tuberc. Other Mycobact. Dis. 15, 100091. doi: 10.1016/j.jctube.2019.100091 31720418 PMC6830119

[B25] Dohal M. Porvaznik I. Solovic I. Mokry J. (2021). Whole genome sequencing in the management of non-tuberculous mycobacterial infections. Microorganisms 9 (11), 2237. doi: 10.3390/microorganisms9112237 34835363 PMC8621650

[B26] Eid J. Fehr A. Gray J. Luong K. Lyle J. Bettman B.. (2009). Real-time DNA sequencing from single polymerase molecules. Science 323 (5910), 133–138. doi: 10.1126/science.1162986 19023044

[B27] Erber J. Weidlich S. Tschaikowsky T. Rothe K. Schmid R. M. Spinner C. D.. (2020). Successful bedaquiline-containing antimycobacterial treatment in post-traumatic skin and soft-tissue infection by *Mycobacterium fortuitum* complex: a case report. BMC Infect. Dis. 20 (1), 1–7. doi: 10.1186/s12879-020-05075-7 PMC7245858 32448204

[B28] Ferrarini M. Moretto M. Ward J. A. Surbanovski N. Stevanovic V. Sargent D. J.. (2013). Anevaluation of the PacBioRS platform for sequencing and denovoassembly of achloroplast genome. BMC Genomics 14 (1), 1–12. doi: 10.1186/1471-2164-14-670 24083400 PMC3853357

[B29] Fitzmaurice A. M. Kolattukudy P. E. (1997). Open reading frame 3, which is adjacent to the mycocerosic acid synthase gene, is expressed as an acyl coenzyme a synthase in *Mycobacterium bovis* BCG. J. @ Bact 179 (8), 2608–2615. doi: 10.1128/jb.179.8.2608-2615.1997 9098059 PMC179010

[B30] Forrellad M. A. Klepp L. I. Gioffre A. Sabio y Garcia J. Morbidoni H. R. Bigi F.. (2013). Virulence factors of the *Mycobacterium tuberculosis* complex. Virulence 4 (1), 3–66. doi: 10.4161/viru.22329 23076359 PMC3544749

[B31] Gharbi R. Khanna V. Frigui W. Mhenni B. Brosch R. Mardassi H. (2021). Phenotypic and genomic hallmarks of a novel, potentially pathogenic rapidly growing mycobacterium species related to the *Mycobacterium fortuitum* complex. Sci. Rep. 11 (1), 1–12. doi: 10.1038/s41598-021-91737-8 34155223 PMC8217490

[B32] Giffin M. M. Raab R. W. Morganstern M. Sohaskey C. D. (2012). Mutational analysis of the respiratory nitrate transporter NarK2 of *Mycobacterium tuberculosis* . PLos One. 7 (9), e45459. doi: 10.1371/journal.pone.0045459 23029022 PMC3445494

[B33] Glickman C. Kammlade S. M. Hasan N. A. Epperson L. E. Davidson R. M. Strong M. (2020). Characterization of integrated prophages within diverse species of clinical nontuberculous mycobacteria. Viro. J. 17(1), 1–13. doi: 10.1186/s12985-020-01394-y PMC7433156 32807206

[B34] Gonzalez-Perez M. Marino-Ramirez L. Parra-Lopez C. A. Murcia M. I. Marquina B. Hernandez-Pando R.. (2013). Virulence and immune response induced by *Mycobacterium avium* complex strains in a model of progressive pulmonary tuberculosis and subcutaneous infection in BALB/c mice. Infect. Immun. 81 (11), 4001–4012. doi: 10.1128/IAI.00150-13 23959717 PMC3811829

[B35] Griffin J. E. Gawronski J. D. DeJesus M. A. Ioerger T. R. Akerley B. J. Sassetti C.M. (2011). High-resolution phenotypic profiling defines genes essential for mycobacterial growth and cholesterol catabolism. PLos Pathog. 7 (9), e1002251. doi: 10.1371/journal.ppat.1002251 21980284 PMC3182942

[B36] Grissa I. Vergnaud G. Pourcel C. (2007). CRISPRFinder: a web tool to identify clustered regularly interspaced short palindromic repeats. Nucleic Acids Res. 35 (suppl_2), W52–W57. doi: 10.1093/nar/gkm360 17537822 PMC1933234

[B37] Groschel M. I. Sayes F. Simeone R. Majlessi L. Brosch R. (2016). ESX secretion systems: mycobacterial evolution to counter host immunity. Nat. Rev. Microbiol. 14 (11), 677–691. doi: 10.1038/nrmicro.2016.131 27665717

[B38] Guo J. Wang C. Han Y. Liu Z. Wu T. Zhang T.. (2016). Identification of lysine acetylation in *Mycobacterium abscessus* using LC–MS/MS after immunoprecipitation. J. Proteome Res. 15 (8), 2567–2578. doi: 10.1021/acs.jproteome.6b00116 27323652

[B39] Han R. Xie D. Tong X. Zhang W. Liu G. Yu N.. (2018). Transcriptomic landscape of *Dendrobium huoshanense* and its genes related to polysaccharide biosynthesis. AGRIS 87 (1), 3574. doi: 10.5586/asbp.3574

[B40] Harris M. A. Clark J. Ireland A. Lomax J. Ashburner M. Hill.. (2004). The gene ontology (GO) database and informatics resource. Nucleic Acids Res. 32 (Database issue), D258–D261. doi: 10.1093/nar/gkh036 14681407 PMC308770

[B41] Hert D. G. Fredlake C. P. Barron A. E. (2008). Advantages and limitations of next-generation sequencing technologies: a comparison of electrophoresis and non-electrophoresis methods. Electrophoresis 29 (23), 4618–4626. doi: 10.1002/elps.200800456 19053153

[B42] Huang Z. Yu K. Xiao Y. Wang Y. Xiao D. Wang D. (2022). Comparative genomic analysis reveals potential pathogenicity and slow-growth characteristics of genus *Brevundimonas* and description of *Brevundimonas pishanensis* sp. nov. Microbiol. Spect. 10 (2), e02468–e02421. doi: 10.1128/spectrum.02468-21 PMC9045160 35416704

[B43] Jia X. Yang L. Li C. Xu Y. Yang Q. Chen F. (2021). Combining comparative genomic analysis with machine learning reveals some promising diagnostic markers to identify five common pathogenic non-tuberculous mycobacteria. Microb. Biotechnol. 14 (4), 1539–1549. doi: 10.1111/1751-7915.13815 34019733 PMC8313281

[B44] Johansen M. D. Kremer L. (2020). CFTR depletion confers hypersusceptibility to *Mycobacterium fortuitum* in a zebrafish model. Front. Cell Infect. Microbiol. 10. doi: 10.3389/fcimb.2020.00357 PMC7396536 32850470

[B45] Jordan T. C. Burnett S. H. Carson S. Caruso S. M. Clase K. Hatfull G. F.. (2014). A broadly implementable research course in phage discovery and genomics for first-year undergraduate students. mBio. 5 (1), e01051–e01013. doi: 10.1128/mBio.01051-13 24496795 PMC3950523

[B46] Kamada K. Yoshida A. Iguchi S. Arai Y. Uzawa Y. Kikuchi K.. (2021). Nationwide surveillance of antimicrobial susceptibility of 509 rapidly growing mycobacteria strains isolated from clinical specimens in Japan. Sci. Rep. 11 (1), 1–10. doi: 10.1038/s41598-021-91757-4 34108590 PMC8190260

[B47] Kanehisa M. Goto S. (2000). KEGG: kyoto encyclopedia of genes and genomes. Nucleic Acids Res. 28 (1), 27–30. doi: 10.1093/nar/28.1.27 10592173 PMC102409

[B48] Katoh K. Standley D. M. (2013). MAFFT multiple sequence alignment software version 7: improvements in performance and usability. Mol. Biol. Evol. 30 (4), 772–780. doi: 10.1093/molbev/mst010 23329690 PMC3603318

[B49] Koh W. J. Kwon O. J. Jeon K. Kim T. S. Lee K. S. Bai G. H.. (2006). Clinical significance of nontuberculous mycobacteria isolated from respiratory specimens in Korea. Chest 129 (2), 341–348. doi: 10.1378/chest.129.2.341 16478850

[B50] Krishnan M. Y. Manning E. J. Collins M. T. (2009). Comparison of three methods for susceptibility testing of *Mycobacterium avium subsp. paratuberculosis* to 11 antimicrobial drugs. J. Antimicrob. Chemother. 64 (2), 310–316. doi: 10.1093/jac/dkp184 19457932

[B51] Kurokawa K. Harada N. Sasano H. Takagi H. Takei S. Takahashi K.. (2020). Pulmonary infection due to fluoroquinolone-resistant *Mycolicibacterium fortuitum*: a case report. BMC Infect. Dis. 20 (1), 1–6. doi: 10.1186/s12879-020-05596-1 PMC7678322 33213390

[B52] Leung K. S. S. Siu G. K. H. Tam K. K. G. To S. W. C. Rajwani R. Yam W. C.. (2017). Comparative genomic analysis of two clonally related multidrug resistant *mycobacterium tuberculosis* by single molecule real-time sequencing. Front. Cell. Infect. Microbiol. 7. doi: 10.3389/fcimb.2017.00478 PMC5694780 29188195

[B53] Li H. Durbin R. (2009). Fast and accurate short read alignment with burrows–wheeler transform. Bioinformatics 25 (14), 1754–1760. doi: 10.1093/bioinformatics/btp324 19451168 PMC2705234

[B54] Lowe T. M. Eddy S. R. (1997). tRNAscan-SE: a program for improved detection of transfer RNA genes in genomic sequence. Nucleic Acids Res. 25 (5), 955–964. doi: 10.1093/nar/25.5.955 9023104 PMC146525

[B55] Luo Z. Hao S. Bai X. Zhang Z. Ma Y. Zhang D.. (2022). Identification and genomic analysis of *Mycobacterium ulcerans* ecovar liflandii from the farmed Chinese tongue sole, *Cynoglossus semilaevis* gunther. Aquaculture 548, 737614. doi: 10.1016/j.aquaculture.2021.737614

[B56] MacGurn J. A. Cox J. S. (2007). A genetic screen for *Mycobacterium tuberculosis* mutants defective for phagosome maturation arrest identifies components of the ESX-1 secretion system. Infect. Immun. 75 (6), 2668–2678. doi: 10.1128/IAI.01872-06 17353284 PMC1932882

[B57] Madacki J. Orgeur M. Mas Fiol G. Frigui W. Ma L. Brosch R. (2021). ESX-1-independent horizontal gene transfer by *Mycobacterium tuberculosis* complex strains. mBio. 12 (3), e00965–e00921. doi: 10.1128/mBio.00965-21 34006663 PMC8262963

[B58] Magrane M. (2011). UniProt knowledgebase: a hub of integrated protein data. Database (Oxford) 2011, bar009. doi: 10.1093/database/bar009 21447597 PMC3070428

[B59] Mandato A. Chai Y. C. (2018). Regulation of antigen 85C activity by reversible s-glutathionylation. IUBMB Life. 70 (11), 1111–1114. doi: 10.1002/iub.1923 30120875

[B60] Maya T. G. Komba E. V. Mensah G. I. Mbelele P. M. Mpagama S. G. Kazwala R. R.. (2022). Drug susceptibility profiles and factors associated with non-tuberculous mycobacteria species circulating among patients diagnosed with pulmonary tuberculosis in Tanzania. PLos One 17 (3), e0265358. doi: 10.1371/journal.pone.0265358 35324922 PMC8947393

[B61] McCarthy A. (2010). Third generation DNA sequencing: pacific biosciences' single molecule real time technology. Chem. Bio. 17 (7), 675–676. doi: 10.1016/j.chembiol.2010.07.004 20659677

[B62] Miotto P. Tessema B. Tagliani E. Chindelevitch L. Starks A. M. Rodwell T. C.. (2017). A standardised method for interpreting the association between mutations and phenotypic drug resistance in *Mycobacterium tuberculosis* . Eur. Respir. J. 50 (6), 1701354. doi: 10.1183/13993003.01354-2017 29284687 PMC5898944

[B63] Morgado S. de Veiga Ramos N. do Nascimento Pereira B. B. Freitas F. da Fonseca E. L. Vicente A. C. (2022a). Multidrug-resistant *Mycolicibacterium fortuitum* infection in a companion cat (*Felis silvestris catus*) in Brazil. Access Microbiol. 4 (2), 317. doi: 10.1099/acmi.0.000317 PMC8941956 35355875

[B64] Morgado S. Ramos N. D. V. Freitas F. da Fonseca E. L. Vicente A. C. (2022b). *Mycolicibacterium fortuitum* genomic epidemiology, resistome and virulome. Mem. Inst. Oswaldo. Cruz. 116, e210247. doi: 10.1590/0074-02760210247 35019071 PMC8752049

[B65] Myers E. W. Sutton G. G. Delcher A. L. Dew I. M. Fasulo D. P. Venter J. C.. (2000). A whole-genome assembly of *Drosophila* . Science 287 (5461), 2196–2204. doi: 10.1126/science.287.5461.2196 10731133

[B66] Nash K. A. Zhang Y. Brown-Elliott B. A. Wallace J. R.J. (2005). Molecular basis of intrinsic macrolide resistance in clinical isolates of *Mycobacterium fortuitum* . J. Anti Chem. 55 (2), 170–177. doi: 10.1093/jac/dkh523 PMC1472656 15590712

[B67] Nawrocki E. P. Burge S. W. Bateman A. Daub J. Eberhardt R. Y. Finn R. D.. (2015). Rfam 12.0: updates to the RNA families database. Nucleic Acids Res. 43 (D1), D130–D137. doi: 10.1093/nar/gku1063 25392425 PMC4383904

[B68] Newton-Foot M. Warren R. M. Sampson S. L. Van Helden P. D. Gey van Pittius N. C. (2016). The plasmid-mediated evolution of the mycobacterial ESX (Type VII) secretion systems. BMC Evo. Bio. 16 (1), 1–12. doi: 10.1186/s12862-016-0631-2 PMC4791881 26979252

[B69] Park S. Suh G. Y. Chung M. P. Kim H. Kwon O. J. Koh W. J.. (2008). Clinical significance of *Mycobacterium fortuitum* isolated from respiratory specimens. Respt. Med. 102 (3), 437–442. doi: 10.1016/j.rmed.2007.10.005 17997087

[B70] Pavlik I. Ulmann V. Weston R. T. (2021). Clinical relevance and environmental prevalence of *Mycobacterium fortuitum* group members. comment on mugetti et al. gene sequencing and phylogenetic analysis: powerful tools for an improved diagnosis of fish mycobacteriosis caused by mycobacterium fortuitum group members. microorganisms 2021, 9, 797. Microorganisms 9 (11), 2345. doi: 10.3390/microorganisms9112345 34835470 PMC8622867

[B71] Price M. N. Dehal P. S. Arkin A. P. (2010). FastTree 2–approximately maximum-likelihood trees for large alignments. PLos One 5 (3), e9490. doi: 10.1371/journal.pone.0009490 20224823 PMC2835736

[B72] Punta M. Coggill P. C. Eberhardt R. Y. Mistry J. Tate J. Finn R. D.. (2012). The pfam protein families database. Nucleic Acids Res. 40 (D1), D290–D301. doi: 10.1093/nar/gkr1065 22127870 PMC3245129

[B73] Qi J. Zheng N. Zhang B. Sun P. Hu S. Li X.. (2013). Mining genes involved in the stratification of Paris polyphylla seeds using high-throughput embryo transcriptome sequencing. BMC Genomics 14 (1), 1–14. doi: 10.1186/1471-2164-14-358 23718911 PMC3679829

[B74] Rabello M. C. D. S. Matsumoto C. K. de Almeida L. G. P. Menendez M. C. de Oliveira R. S. Leao S. C.. (2012). First description of natural and experimental conjugation between mycobacteria mediated by a linear plasmid. PLos One 7 (1), e29884. doi: 10.1371/journal.pone 22235347 PMC3250492

[B75] Ramon-Garcia S. Otal I. Martin C. Gomez-Lus R. Ainsa J. A. (2006). Novel streptomycin resistance gene from *Mycobacterium fortuitum* . Antimicrob. Agents Chemother. 50 (11), 3920–3922. doi: 10.1128/AAC.00223-06 16954315 PMC1635185

[B76] Reves R. Schluger N. W. (2014). Update in tuberculosis and nontuberculous mycobacterial infections 2013. Am. J. Respir. Crit. Care Med. 189 (8), 894–898. doi: 10.1164/rccm.201402-0210UP 24735031

[B77] Richard M. Gutierrez A. V. Viljoen A. Rodriguez-Rincon D. Roquet-Baneres F. Kremer L.. (2019). Mutations in the *MAB_2299c* TetR regulator confer cross-resistance to clofazimine and bedaquiline in *Mycobacterium abscessus* . Antimicrobial Agents chemother. 63 (1), e01316–e01318. doi: 10.1128/AAC.01316-18 PMC6325171 30323043

[B78] Robbertse B. Strope P. K. Chaverri P. Gazis R. Ciufo S. Schoch C. L.. (2017). Improving taxonomic accuracy for fungi in public sequence databases: applying ‘one name one species’ in well-defined genera with trichoderma/hypocrea as a test case. Database (Oxford) 2017, bax072. doi: 10.1093/database/bax072 29220466 PMC5641268

[B79] Roberts R. J. Carneiro M. O. Schatz M. C. (2013). The advantages of SMRT sequencing. Gen. Biol. 14 (6), 1–4. doi: 10.1186/gb-2013-14-6-405 PMC3953343 23822731

[B80] Samten B. Wang X. Barnes P. F. (2009). *Mycobacterium tuberculosis* ESX-1 system-secreted protein ESAT-6 but not CFP10 inhibits human T-cell immune responses. Tuberculosis 89 (Suppl 1), S74–S76. doi: 10.1016/S1472-9792(09)70017-4 20006311

[B81] Sassetti C. M. Boyd D. H. Rubin E. J. (2003). Genes required for mycobacterial growth defined by high density mutagenesis. Mol. Microbiol. 48 (1), 77–84. doi: 10.1046/j.1365-2958.2003.03425.x 12657046

[B82] Saxena S. Spaink H. P. Forn-Cuni G. (2021). Drug resistance in nontuberculous mycobacteria: mechanisms and models. Biology 10 (2), 96. doi: 10.3390/biology10020096 33573039 PMC7911849

[B83] Schon T. Werngren J. Machado D. Borroni E. Wijkander M. Cambau E.. (2020). Antimicrobial susceptibility testing of *Mycobacterium tuberculosis* complex isolates–the EUCAST broth microdilution reference method for MIC determination. Clinc. Microbiol. Infect. 26 (11), 1488–1492. doi: 10.1016/j.cmi.2020.07.036 32750539

[B84] Senhaji-Kacha A. Esteban J. Garcia-Quintanilla M. (2021). Considerations for phage therapy against *Mycobacterium abscessus* . Front. Microbiol. 11. doi: 10.3389/fmicb.2020.609017 PMC7847891 33537013

[B85] Sewell E. (2017). Characterizing the intact prophage of *Mycobacterium chelonae bergey* . Honors College 265. Available at: https://digitalcommons.library.umaine.edu/honors/265

[B86] Sirakova T. D. Fitzmaurice A. M. Kolattukudy P. (2002). Regulation of expression of mas and *fadD28*, two genes involved in production of dimycocerosyl phthiocerol, a virulence factor of *Mycobacterium tuberculosis* . J. Bacteriol. 184 (24), 6796–6802. doi: 10.1128/JB.184.24.6796-6802.2002 12446629 PMC135475

[B87] Smith J. Manoranjan J. Pan M. Bohsali A. Xu J. Gao L. Y.. (2008). Evidence for pore formation in host cell membranes by ESX-1-secreted ESAT-6 and its role in *Mycobacterium marinum* escape from the vacuole. Infect. Immun. 76 (12), 5478–5487. doi: 10.1128/IAI.00614-08 18852239 PMC2583575

[B88] Stanke M. Schoffmann O. Morgenstern B. Waack S. (2006). Gene prediction in eukaryotes with a generalized hidden Markov model that uses hints from external sources. BMC Bioinf. 7 (1), 1–11. doi: 10.1186/1471-2105-7-62 PMC1409804 16469098

[B89] Sun Q. Yan J. Liao X. Wang C. Wang C. Pan J.. (2022). Trends and species diversity of nontuberculous mycobacteria isolation from respiratory samples in northern china 2014–2021. Front. Public Health 10. doi: 10.3389/fpubh.2022.923968 PMC9341428 35923959

[B90] Tatusov R. L. Fedorova N. D. Jackson J. D. Jacobs A. R. Kiryutin B. Natale D. A.. (2003). The COG database: an updated version includes eukaryotes. BMC Bioinf. 4, 41. doi: 10.1186/1471-2105-4-41 PMC222959 12969510

[B91] Teng J. L. Yeung M. L. Chan E. Jia L. Lin C. H. Woo P. C.. (2017). PacBio but not illumina technology can achieve fast, accurate and complete closure of the high GC, complex *Burkholderia pseudomallei* two-chromosome genome. Front. Microbiol. 8. doi: 10.3389/fmicb.2017.01448 PMC5539568 28824579

[B92] Treangen T. J. Salzberg S. L. (2011). Repetitive DNA and next-generation sequencing: computational challenges and solutions. Nat. Rev. Genet. 13 (1), 36–46. doi: 10.1038/nrg3117 22124482 PMC3324860

[B93] Velmurugan K. Chen B. Miller J. L. Azogue S. Gurses S. Briken V.. (2007). *Mycobacterium tuberculosis nuoG* is a virulence gene that inhibits apoptosis of infected host cells. PLos Pathog. 3 (7), e110. doi: 10.1371/journal.ppat.0030110 17658950 PMC1924871

[B94] Wang K. (2020). ANNOVAR documentation. Available at: https://annovar.openbioinformatics.org/en/latest/

[B95] Wang J. Haapalainen M. Schott T. Thompson S. M. Smith G. R. Pirhonen M.. (2017). Genomic sequence *of 'Candidatus liberibacter* solanacearum haplotype c and its comparison with haplotype a and b genomes. PLos One 12 (2), e0171531. doi: 10.1371/journal.pone.0171531 28158295 PMC5291501

[B96] Weber I. Fritz C. Ruttkowski S. Kreft A. Bange F. C. (2000). Anaerobic nitrate reductase (narGHJI) activity of *Mycobacterium bovis* BCG *in vitro* and its contribution to virulence in immunodeficient mice. Mol. Microbiol. 35 (5), 1017–1025. doi: 10.1046/j.1365-2958.2000.01794.x 10712684

[B97] Wee W. Y. Tan T. K. Jakubovics N. S. Choo S. W. (2016). Whole-genome sequencing and comparative analysis of *Mycobacterium brisbanense* reveals a possible soil origin and capability in fertiliser synthesis. PLos One 11 (3), e0152682. doi: 10.1371/journal.pone.0152682 27031249 PMC4816395

[B98] Winthrop K. L. Marras T. K. Adjemian J. Zhang H. Wang P. Zhang Q. (2020). Incidence and prevalence of nontuberculous mycobacterial lung disease in the large US managed care health plan 2008–2015. Ann. Am. Thorac. Soc 17 (2), 178–185. doi: 10.1513/AnnalsATS.201804-236OC 31830805 PMC6993793

[B99] Yang J. Chen L. Sun L. Yu J. Jin Q. (2007). VFDB 2008 release: an enhanced web-based resource for comparative pathogenomics. Nucleic Acids Res. 36 (suppl_1), D539–D542. doi: 10.1093/nar/gkm951 17984080 PMC2238871

[B100] Yang H. Wang K. (2015). Genomic variant annotation and prioritization with ANNOVAR and ANNOVAR. Nat. Protoc. 10 (10), 1556–1566. doi: 10.1038/nprot.2015.105 26379229 PMC4718734

[B101] Yoon S. H. Ha S. M. Lim J. Kwon S. Chun J. (2017). A large-scale evaluation of algorithms to calculate average nucleotide identity. Antonie Van Leeuwenhoek 110 (10), 1281–1286. doi: 10.1007/s10482-017-0844-4 28204908

[B102] Zhang Y. J. Reddy M. C. Ioerger T. R. Rothchild A. C. Dartois V. Rubin E. J.. (2013). Tryptophan biosynthesis protects mycobacteria from CD4 T-cell-mediated killing. Cell 155 (6), 1296–1308. doi: 10.1016/j.cell.2013.10.045 24315099 PMC3902092

[B103] Zhang F. Xie J. P. (2011). Mammalian cell entry gene family of *Mycobacterium tuberculosis* . Mol. Cell Biochem. 352 (1), 1–10. doi: 10.1007/s11010-011-0733-5 21258845

[B104] Zhu L. Zhong J. Jia X. Liu G. Kang Y. Chen F.. (2016). Precision methylome characterization of *Mycobacterium tuberculosis* complex (MTBC) using PacBio single-molecule real-time (SMRT) technology. Nucleic Acids Res. 44 (2), 730–743. doi: 10.1093/nar/gkv1498 26704977 PMC4737169

