# Supplementary material for: Comparative genome analysis reveals high-level drug resistance markers in a clinical isolate of Mycobacterium fortuitum subsp. fortuitum MF GZ001
Source: Front Cell Infect Microbiol. 2023 Jan 4;12:1056007. doi: 10.3389/fcimb.2022.1056007 (PMC9846761; doi:10.3389/fcimb.2022.1056007)
Supplement: Supplementary file 1 [file DataSheet_1.zip › Data Sheet.pdf]

Supplementary materials

Figure S1 Relationship analysis between GC content and sequencing depth.
Figure S2 Pathway categories of genes by KEGG annotation of MF strain, whereas X-axis indicates the number of genes and; the Y-axis indicates pathways.
Figure S3 COG functional classification of genes of MF strain, whereas X-axis indicates COG class; Y-axis indicates gene number in the COG term.
Figure S4 The non-redundant (NR) species distribution map. Note: The pie chart reflects the distribution ratio of different species, and different colors represent different species.
Figure S5 Bar plot of genes in GO Terms. Y-axis is the GO term; the X-axis represents the number of genes.
Figure S6 CAZy classification of genes. Notes: X-axis is CAZy class; the Y-axis is the gene number of CAZy class.
Figure S7 SNP/INDE distribution map of identified MF strain compared with reference strain and other distantly related strains.
Figure S8 Phylogenetic analysis. (A) Construction of phylogenetic tree based on 16S rRNA and (B) *hsp65;* (C) Construction of Supermatrix based on *rpoB*, *16S rRNA*, and *hsp65*. Phylogenetic tree results suggest the MF GZ001 strain is a new member of the *M. fortuitum* complex.
Table S1 Preliminary statistical analysis of the raw data obtained by sequencing.
Table S2 Statistics of clean data after trimming.
Table S3 Data retention statistics of clean data after quality control.
Table S4 Quality evolution of PacBio reads statistics using single-molecule real-time (SMRT) sequencing technology.
Table S5 Genome assembly statistics of *M. fortuitum* clinical strains generated by using single-molecule real-time (SMRT) sequencing.
Table S6 Mapping statistics to determine the sequencing depth.
Table S7 Prophage prediction result statistics are shown in the following table.
Table S8 The CRISPR prediction result is shown in the following table.
Table S9 Summary of gene annotation result statistics.
Table S10 drug resistance-related genes distribution in MF GZ001strain.
Table S11 Drug susceptibility testing of MF GZ001isolate.
Table S12 All Mycobacterial strains were used in this study.
Table S13 SNPs in drug resistance genes analyzed in the MF GZ001 genome.
Table S14 Predicted virulence genes analysis across the MF GZ001 and 21 other mycobacterial genomes.
