# Supplementary material for: Comparative genome analysis reveals high-level drug resistance markers in a clinical isolate of Mycobacterium fortuitum subsp. fortuitum MF GZ001
Source: Front Cell Infect Microbiol. 2023 Jan 4;12:1056007. doi: 10.3389/fcimb.2022.1056007 (PMC9846761; doi:10.3389/fcimb.2022.1056007)
Supplement: Supplementary file 1 [file DataSheet_1.zip › Figure S3 .pdf]

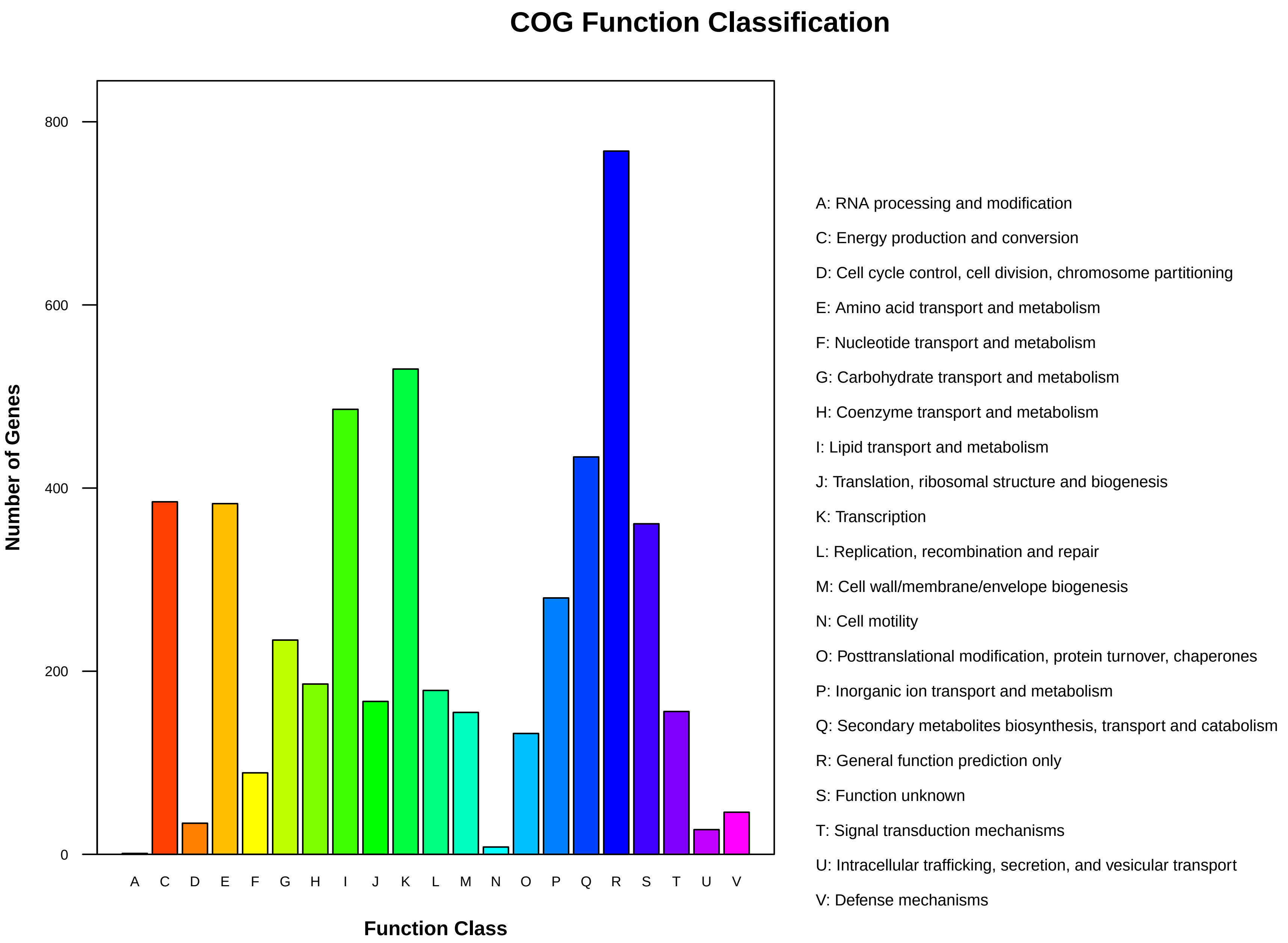
COG Function Classification
Number of Genes
800
600
400
200
0
A C D E F G H I J K L M N O P Q R S T U V
Function Class
A: RNA processing and modification
C: Energy production and conversion
D: Cell cycle control, cell division, chromosome partitioning
E: Amino acid transport and metabolism
F: Nucleotide transport and metabolism
G: Carbohydrate transport and metabolism
H: Coenzyme transport and metabolism
I: Lipid transport and metabolism
J: Translation, ribosomal structure and biogenesis
K: Transcription
L: Replication, recombination and repair
M: Cell wall/membrane/envelope biogenesis
N: Cell motility
O: Posttranslational modification, protein turnover, chaperones
P: Inorganic ion transport and metabolism
Q: Secondary metabolites biosynthesis, transport and catabolism
R: General function prediction only
S: Function unknown
T: Signal transduction mechanisms
U: Intracellular trafficking, secretion, and vesicular transport
V: Defense mechanisms
