# Supplementary material for: Comparative genome analysis reveals high-level drug resistance markers in a clinical isolate of Mycobacterium fortuitum subsp. fortuitum MF GZ001
Source: Front Cell Infect Microbiol. 2023 Jan 4;12:1056007. doi: 10.3389/fcimb.2022.1056007 (PMC9846761; doi:10.3389/fcimb.2022.1056007)

A

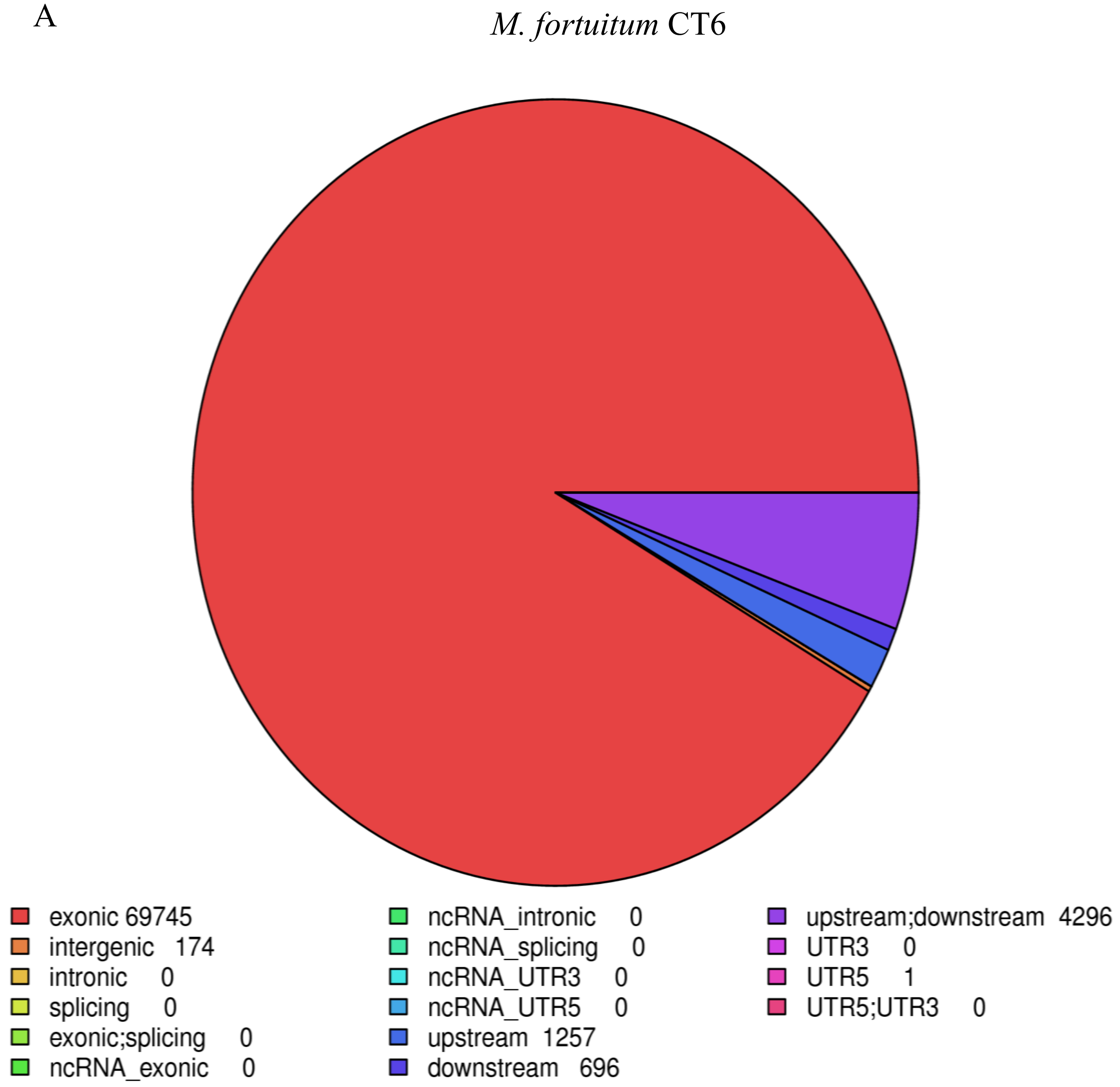

M. fortuitum CT6
exonic 69745
intergenic 174
intronic 0
splicing 0
exonic;splicing 0
ncRNA_exonic 0
ncRNA_intronic 0
ncRNA_splicing 0
ncRNA_UTR3 0
ncRNA_UTR5 0
upstream 1257
downstream 696
upstream;downstream 4296
UTR3 0
UTR5 1
UTR5;UTR3 0

B

M. abscessus GZ002

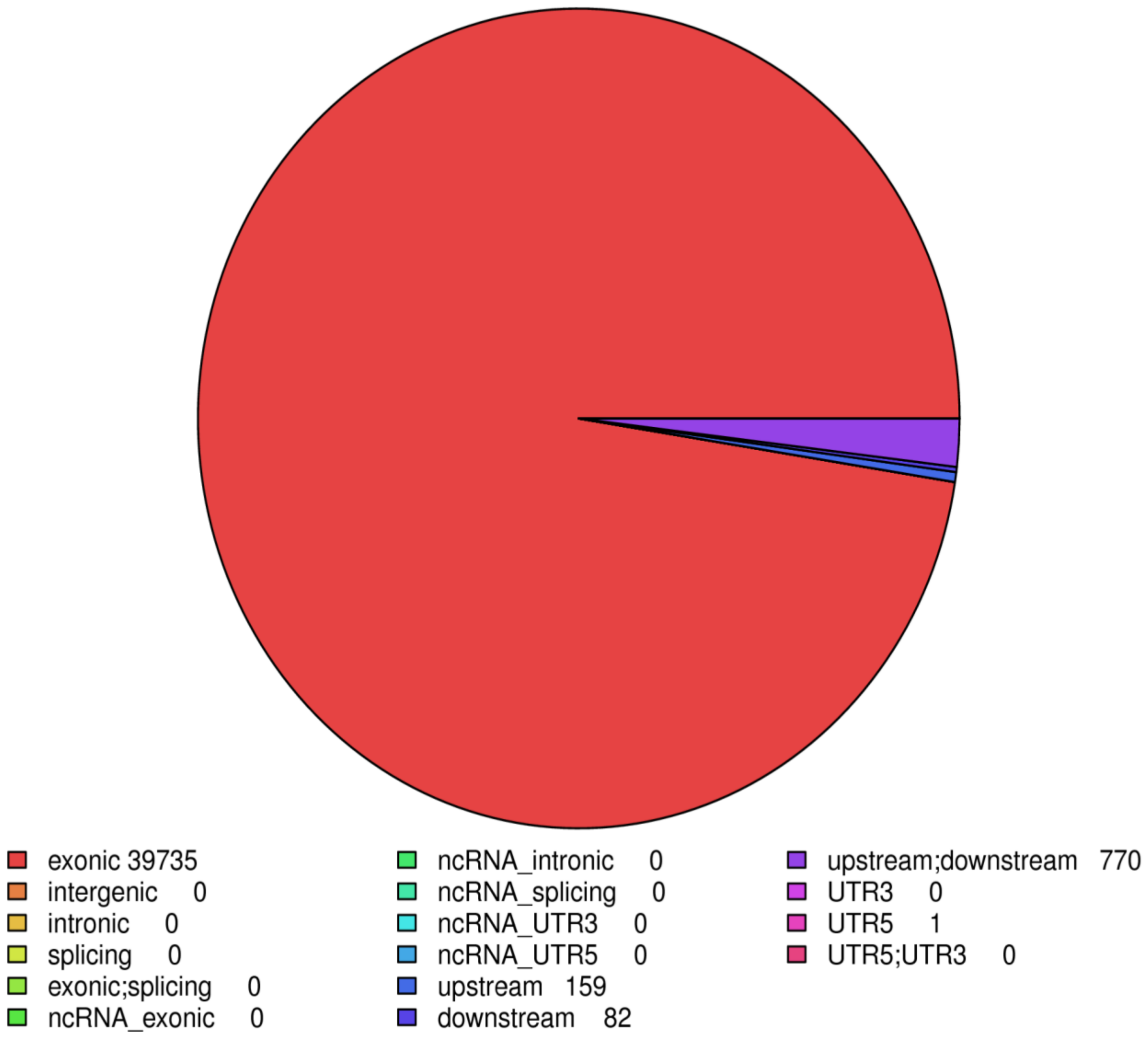

C

M. smegmatis C^2 155

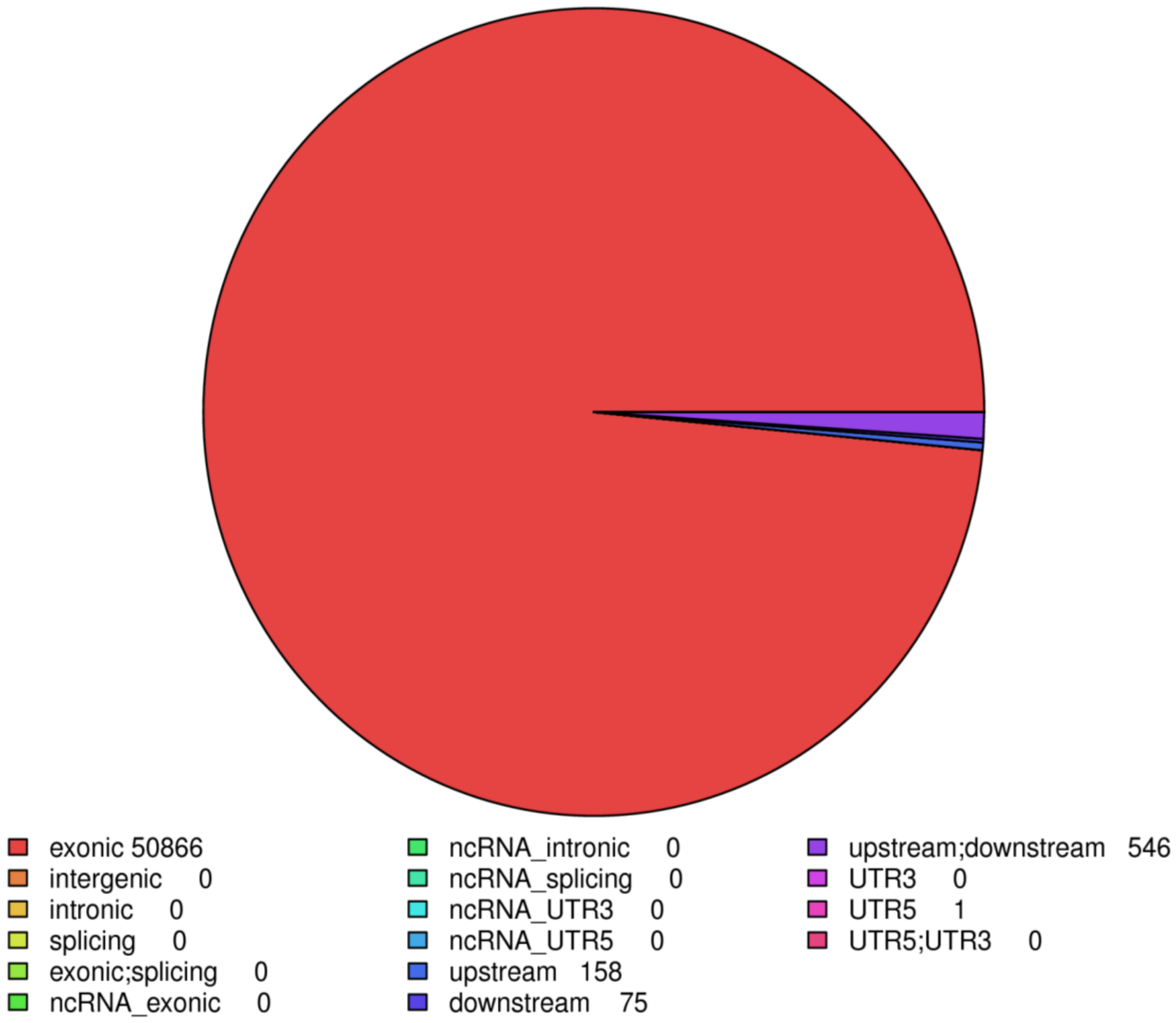

D *M. tuberculosis* H37Rv

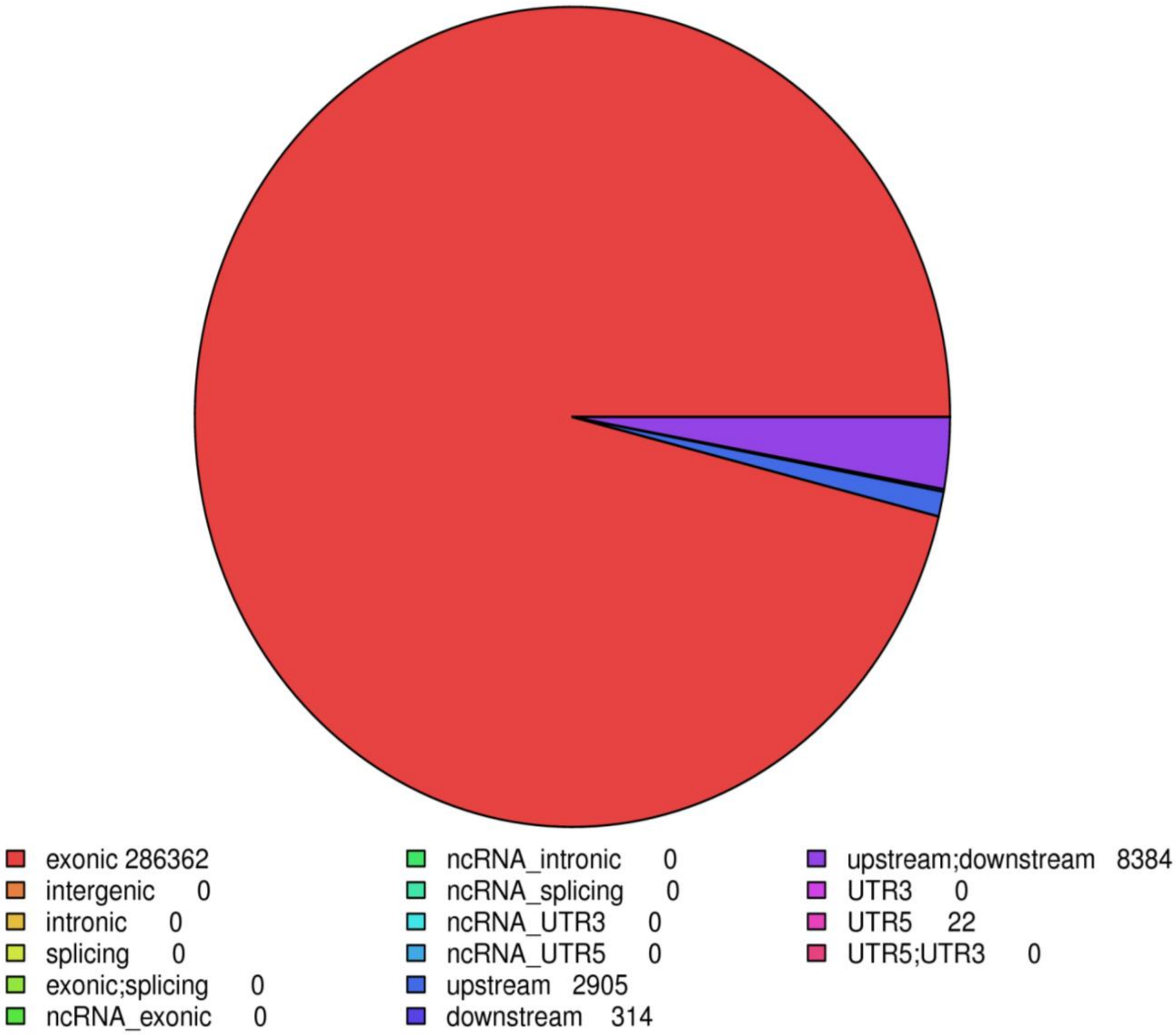

Supplement: Supplementary file 1 [file DataSheet_1.zip › Figure S7.pdf]
