# Supplementary material for: Comparative genome analysis reveals high-level drug resistance markers in a clinical isolate of Mycobacterium fortuitum subsp. fortuitum MF GZ001
Source: Front Cell Infect Microbiol. 2023 Jan 4;12:1056007. doi: 10.3389/fcimb.2022.1056007 (PMC9846761; doi:10.3389/fcimb.2022.1056007)
Supplement: Supplementary file 1 [file DataSheet_1.zip › Supplementary materials 1.pdf]

Materials and Methods

Illumina sequencing library construction and bioinformatics analysis

Next-generation sequencing library preparations were constructed following the manufacturer’s protocol. 200 μg genomic DNA was randomly fragmented by Covaris to an average size of 300-350 bp. The fragments were treated with end prep enzyme mix for end repairing, 5’ phosphorylation, and 3’ adenylated, to add adaptors to both ends. Size selection of adaptor-ligated DNA was then performed by DNA Cleanup beads. Each sample was then amplified by PCR for 8 cycles using P5 and P7 primers, with both primers carrying sequences that can anneal with flow cell to perform bridge PCR and the P7 primer carrying a six-base index allowing for multiplexing. The PCR products were cleaned up and validated using an Agilent 2100 Bioanalyzer. The qualified libraries were sequenced pair-end PE150 on the Illumina HiseqXten/Novaseq/MGI2000 System. For the PacBio sequencing library, 5-10 μg genomic DNA was sheared into 10-15 Kb fragments using a g-TUBE device. Then the library was constructed using the SMRTbell® Express Template Preparation Kit 2.0. Briefly, the sheared fragments were through single-strand overhang removal, DNA damage repair, end repair, A-tailing, barcoded overhang adapters ligation. The library was quantified using Qubit 3.0 Fluorometer (Invitrogen, Carlsbad, CA), and the size of the library was checked using an Agilent 2100 Bioanalyzer System. Subsequent steps were followed as per the manufacturer’s instructions to prepare the SMRTbell library.

The library was sequenced using the Sequel II sequencing platform (McCarthy et al., 2010). PacBio reads were assembled using HGAP4/Falcon of WGS-Assembler 8.2 (Myers et al., 2000; Venter et al., 2001; Istrail et al., 2004; Levy et al., 2007; Goldberg et al., 2006; Berlin et al., 2015). And then we re-corrected the genome with the software Pilon using previous Illumina data or Quiver using Pacbio reads. The Prodigal (Delcher et al., 2007) /Augustus (Stanke et al., 2006) gene-finding software has been used for finding coding genes. Transfer RNAs (tRNAs) were detected in the genome using the program tRNAscan-SE (Lowe et al., 1997) with default parameter settings. rRNA was identified by using Barrnap. Other RNAs were identified with the help of the rfam database. The coding genes were annotated with National Center for Biotechnology Information (NCBI) NR database by BLAST (screening condition is displayed in Table1.1. Then the functions of genes were annotated by GO) (Gene Ontology) (Gene Ontology Consortium 2004; Ogata et al., 2004 database, and the pathways were annotated using the KEGG (Kyoto Encyclopedia of Genes and Genomes) (Kanehisa et al., 2000) database. The proteins encoded by genes were classified on a phylogenetic classification by the database of COG/KOG (Clusters of Orthologous Groups) (Tatusov et al., 1997).

Table1.1 Annotation screening condition

**DataBase**	**Software**	**Screening index**	**Result number**
NR	Diamond blastp	E-value <0.00001,other arguments default	Best 1
KEGG	Blastn	E-value <0.00001,other arguments default	Best 1
GO	interproscan	Default parameters	Best 1
COG/KOG	rpstblastn	E-value <0.00001,other arguments default	Best 1

CAZy …	Diamond blastp	E-value <0.00001,other arguments default	Best 1

Note: "…" is TCDB, CARD, Mvirdb, PHI, VFDB Pfam, SwissProt database

References

Berlin, K., Koren, S., Chin, C. S., Drake, J. P., Landolin, J. M., and Phillippy, A. M. (2015). Assembling large genomes with single-molecule sequencing and locality-sensitive hashing. *Nat. Biotechnol.* 33 (6), 623-630. doi: 10.1038/nbt.3238

Delcher, A. L., Bratke, K. A., Powers, E. C., and Salzberg, S. L. (2007). Identifying bacterial genes and endosymbiont DNA with Glimmer. *Bioinformatics* 23 (6), 673-679. doi: 10.1093/bioinformatics/btm009

Gene Ontology Consortium. (2004). The Gene Ontology (GO) database and informatics resource. *Nucleic Acids Res.* 32 (suppl_1), D258-D261. doi: 10.1093/nar/gkh036

Goldberg, S. M., Johnson, J., Busam, D., Feldblyum, T., Ferriera, S., Venter, J. C., et al. (2006). A Sanger/pyrosequencing hybrid approach for the generation of high-quality draft assemblies of marine microbial genomes. *Proc. Natl. Acad. Sci.* 103 (30), 11240-11245. doi: 10.1073/pnas.0604351103

Istrail, S., Sutton, G. G., Florea, L., Halpern, A. L., Mobarry, C. M., Venter, J. C., et al. (2004). Whole-genome shotgun assembly and comparison of human genome assemblies. *Proc. Natl. Acad. Sci.* 101 (7), 1916-1921. doi: 10.1073/pnas.0307971100

Kanehisa, M., and Goto, S. (2000). KEGG: kyoto encyclopedia of genes and genomes. *Nucleic Acids Res.* 28 (1), 27-30. doi: 10.1093/nar/28.1.27

Levy, S., Sutton, G., Ng, P. C., Feuk, L., Halpern, A. L., Venter, J. C., et al. (2007). The diploid genome sequence of an individual human. *PLoS Biol.* 5 (10), e254. doi: 10.1371/journal.pbio.0050254

Lowe, T. M., and Eddy, S. R. (1997). tRNAscan-SE: a program for improved detection of transfer RNA genes in genomic sequence. *Nucleic Acids Res.* 25 (5), 955-964. doi: 10.1093/nar/25.5.955

McCarthy, A. (2010). Third generation DNA sequencing: pacific biosciences' single molecule real-time technology. *Chem. Biol.* 17 (7), 675-676. doi: 10.1016/j.chembiol.2010.07.004

Myers, E. W., Sutton, G. G., Delcher, A. L., Dew, I. M., Fasulo, D. P., Venter, J. C. et al. (2000). A whole-genome assembly of Drosophila. *Science* 287 (5461), 2196-2204. doi: 10.1126/science.287.5461.219

Ogata, H., Goto, S., Sato, K., Fujibuchi, W., Bono, H., and Kanehisa, M. (1999). KEGG: Kyoto encyclopedia of genes and genomes. *Nucleic Acids Res.* 27 (1), 29-34. doi: 10.1093/nar/27.1.29

Stanke, M., Schoffmann, O., Morgenstern, B., and Waack, S. (2006). Gene prediction in eukaryotes with a generalized hidden Markov model that uses hints from external sources. *BMC Bioinformatics* 7 (1), 1-11. doi: 10.1186/1471-2105-7-62

Tatusov, R. L., Koonin, E. V., and Lipman, D. J. (1997). A genomic perspective on protein families. *Science* 278 (5338), 631-637. doi: 10.1126/science.278.5338.6

Venter, J. C., Adams, M. D., Myers, E. W., Li, P. W., Mural, R. J., Kalush, F., et al. (2001). The sequence of the human genome. *Science* 91 (5507), 1304-1351. doi: 10.1126/science.1058040
