# Supplementary material for: Comparative genome analysis reveals high-level drug resistance markers in a clinical isolate of Mycobacterium fortuitum subsp. fortuitum MF GZ001
Source: Front Cell Infect Microbiol. 2023 Jan 4;12:1056007. doi: 10.3389/fcimb.2022.1056007 (PMC9846761; doi:10.3389/fcimb.2022.1056007)
Supplement: Supplementary file 1 [file DataSheet_1.zip › Supplementary Tables .docx]

Table S1 Preliminary statistical analysis of the raw data obtained by sequencing

| Sample | Average read average length (bp) | Total number of sequencing read count | Total number of sequencing base count | The percentage of bases with Phared value greater than 20 or 30 | | GC (%) | Undetermined base count per million bases  N (ppm) |
| --- | --- | --- | --- | --- | --- | --- | --- |
|  |  |  |  | Q20 (%) | Q30 (%) |  |  |
| MF GZ001 | 150.00 | 19187140 | 2878071000 | 98.27 | 95.13 | 67.37 | 6.38 |

Table S2 Statistics of clean data after trimming

| Sample | Average length of reads | Total number of reads | Total number of bases | Percentage of bases that have less than 1% probability of error Q20 (%) | Percentage of bases that have less than 0.1% probability of error Q30 (%) | GC (%) | Undetermined base count per million bases  N(ppm) |
| --- | --- | --- | --- | --- | --- | --- | --- |
| MF GZ001 | 149.50 | 19164502 | 2865001515 | 98.36 | 95.23 | 67.47 | 2.42 |

Table S3 Data retention statistics of clean data after quality control

| Sample | The number of raw data PE reads | The number of clean reads after quality control | The ratio of Reads (%)  (Percentage of clean reads out of PF Reads) | PF Bases (bp)  (The number of raw data bases count) | Clean Bases (bp)  (The number of clean bases after quality control ) | The ratio of bases (%)  (Clean bases percentage of PF bases) |
| --- | --- | --- | --- | --- | --- | --- |
| MF GZ001 | 19187140 | 19164502 | 99.88 | 2878071000 | 2865001515 | 99.55 |

Table S4 Quality evolution of PacBio reads statistics using single-molecule real-time (SMRT) sequencing technology

| Sample  name | Sequences： number of reads | Bases： number of reads | Minimum length of reads | The maximum length of reads | Average length of reads | N50 | (C+G)% | Ns% |
| --- | --- | --- | --- | --- | --- | --- | --- | --- |
| MF GZ001 | 1368432 | 4062640505 | 51 | 61689 | 2968.83 | 3362 | 64.41 | 0.00 |

Table S5 Genome assembly statistics of *M. fortuitum* GZ001 clinical strains generated by using single-molecule real-time (SMRT) sequencing

| Sample  name | Sequences： number of reads | Bases： number of reads | Minimum length of reads | The maximum length of reads | The average length of reads | N50 | (C+G)% | Ns% |
| --- | --- | --- | --- | --- | --- | --- | --- | --- |
| MF GZ001 | 1 | 6413573 | 6413573 | 6413573 | 6413573.00 | 6413573 | 66.18 | 0.00 |

Table S6 Mapping statistics to determine the sequencing depth

| Sample Name | Total Reads: Number of sequences for mapping | Reads mapped to the genome： Number of sequences mapped | Mapped Reads Ratio(%)： Mapping ratio | Coverage(%): Reads coverage on genome | Mean depth： The Mean depth of reads mapped on the genome |
| --- | --- | --- | --- | --- | --- |
| MF GZ001 | 1368432 | 1351925 | 98.79 | 100.00 | 574.33 |

Table S7 Prophage prediction result statistics are shown in the following table

| Sample | Prophage number | Total prophage length | Average length | *attL* length | *attR* length |
| --- | --- | --- | --- | --- | --- |
| MF GZ001 | 1 | 39521 bp (5079685 to 5119205) | 39521.00 bp | 13 bp (5081949 to 5081962) | 13 bp (5119651 to 5119664) |

Table S8 CRISPR prediction result is shown in the following table

| Sample | Seq_ID | Start site | End site | Repeat number |
| --- | --- | --- | --- | --- |
| MF GZ001 | MF_pilon | 1223770 | 1223874 | 2 |
| MF GZ001 | MF_pilon | 2809721 | 2809816 | 2 |
| MF GZ001 | MF_pilon | 5505875 | 5506121 | 3 |

Table S9 Summary of gene annotation result statistics

| Sample | Gene number | NR | KEGG | GO | COG | CAZy | Pfam | Swiss_Prot |
| --- | --- | --- | --- | --- | --- | --- | --- | --- |
| MF GZ001 | 6156 | 6133 | 2411 | 3464 | 4315 | 467 | 4962 | 4296 |

Table S10 Drug resistance-related genes distribution in MF GZ001strain

| Drug class | triclosan | macrolides antibiotic | FQs | TET antibiotic | penam | cephalosporin | Peptide  antibiotics | phenicol antibiotic | cephamycin | RIF  antibiotics |
| --- | --- | --- | --- | --- | --- | --- | --- | --- | --- | --- |
| No. of genes | 71 | 60 | 47 | 45 | 38 | 28 | 27 | 24 | 21 | 21 |

Note: Fluoroquinolone; FQs, tetracycline; TET.

Table S11 Drug susceptibility testing of MF GZ001isolate

| Drug Names | MICs (µg/mL) value |
| --- | --- |
| RFB | 4 |
| IMP | 64 |
| RIF | 64 |
| VAN | 32 |
| STR | 64 |
| AMK | 8 |
| CLA | 8 |
| EMB | 4 |
| INH | 8 |
| SMZ | >128 |
| LZD | 32 |
| CLF | >128 |
| LEV | 16 |
| Carbapenems | >128 |

RFB: Rifabutin, IMP: Imipenem, RIF: Rifampicin, VAN: Vancomycin, STR: Streptomycin, AMK: Amikacin, CLA: Clarithromycin, EMB: Ethambutul, INH: Isoniazid, SMZ: Sulfamethoxazole, LZD: Linezolid, CLF: Clofazimine. LEV: Levofloxacin

Table S12 All Mycobacterial strains used in this study

| Strains name |
| --- |
| *M. fortuitum :* Identified strain MF GZ001 |
| *Mycolicibacterium cosmeticum* |
| *M. tuberculosis* H37Rv |
| *M. abscessus* GZ002 |
| *M. smegmatis* C^2^ 155 |
| *Mycobacterium fortuitum* DSM46621 |
| *Mycobacterium fortuitum* CT6 |
| *Mycobacterium fortuitum* W4 |
| Mycobacterium peregrinum CIP 105382 |
| Mycobacterium alvei CIP103464 |
| Mycobacterium septicum DSM44393 |
| *Mycobacterium pallens* |
| [*Mycobacteroides abscessus subsp. bolletii*](https://www.ncbi.nlm.nih.gov/genome/3639?genome_assembly_id=1566358) |
| M.SP.KMS |
| M.sp.MCS |
| *Mycobacterium diernhoferi* |
| *Mycobacterium florentinum* |
| *Mycolicibacterium bacteremicum* |
| *Mycolicibacterium neoaurum* |
| *Mycolicibacterium brisbanense* |
| *Mycobacterium aubagnesnse* |
| M. phocaicum **DSM 45104** |
| M. mucogenicum **DSM 44124** |
| *Mycobacterium boenickei* |
| *Mycobacterium conceptionense* |
| *Mycobacterium fortuitum subsp acetamidolyticum* |
| *Mycobacterium farcinogennese* |
| *Mycobacterium senegaiense* |
| *Mycobacterium houstonense* |
| *Mycobacterium canettii* |
| *Mycobacterium moriokaense* |
| *Mycobacterium fallax* |
| *Mycobacterium wolinsky* |
| *Mycobacterium mageritense* |
| *Mycobacterium rhodesiae* |
| *Mycobacterium insubricum* |
| *Mycobacterium sphagni* |
| *Mycobacterium chubuense* |
| *Mycobacterium leprae* |
| *Mycobacterium psychrotoloerance* |
| *Mycobacterium duvalii* |
| *Mycolicibacterium fortuitum sbsp acetamidalyticum* |
| *Mycolicibacterium farcinogenes* |
| *Mycobacterium marinum* |
| [*Mycolicibacterium peregrinum*](https://www.ncbi.nlm.nih.gov/genome/40808?genome_assembly_id=488246) |
| *Mycobacterium porcinum* |
| [*Mycobacteroides abscessus subsp. bolletii*](https://www.ncbi.nlm.nih.gov/genome/3639?genome_assembly_id=592669) |
| *M. gilvum* |
| *M. phlei* |
| *Mycolicibacterium intracellure* ATCC |
| *Mycolicibacterium avium* |
| *Mycolicibacterium senegaliense* |
| *Mycobacterium bovis* BCG |
| *M. vanbaalenii* |
| *M. tusciae* |
| *Mycobacterium tusciae* CIP |
| *M. ulcerans* |
| *M.* sp JLS |
| *Mycobacterium intracellulare subsp. intracellulare* |
| *M. kansasii* |
| *M. parascrofulaceum* |
| *M. hassiacum* |

Table S13 SNPs in drug resistance genes analyzed in the MF GZ001 genome

| Drug  classes | MF GZ001 number | Ref. | Alternation | Gene names | Resistance  mechanisms | Descriptions |
| --- | --- | --- | --- | --- | --- | --- |
| FQs, aminocoumarin | 1_6 | A | G | *gyrB* | antibiotic target alteration | A point mutation in *M. tuberculosis gyrB* resulting in FQs resistance |
| FQs, neomycin | 1_7 | T | C | g*yrA* | antibiotic target alteration | Point mutation of *M. tuberculosis* *gyrA* resulted in the lowered affinity between FQs and *gyrA*. Thus, conferring resistance. |
| aminoglycoside (STR) | 1_422 | C | T | *AAC(2')-Ib* | antibiotic inactivation | *AAC(2')-Ib* is a chromosomal-encoded aminoglycoside acetyltransferase in *M. fortuitum* and *A. baumannii* |
| peptide antibiotic | 1_446 | - | C | *vph* | antibiotic inactivation | *vph* is a phosphotransferase that confers resistance to viomycin in *Streptomyces vinaceus* |
| polyamine | 1_668 | G | C | *iniA* | antibiotic target alteration | Specific mutations in *M. tuberculosis* *iniA* result in resistance to EMB |
| polyamine | 1_669 | A | G | *iniC* | antibiotic efflux | Specific mutations that occur on *M. tuberculosis iniC* cause it to be ethambutol resistant. |
| phenicol antibiotic | 1_1252 | - | C | *cmx* | antibiotic efflux | Transposon-encoded chloramphenicol exporter is found in *Corynebacterium* striatum and *Pseudomonas aeruginosa.* |
| Peptide, RIF | 1_1356 | - | CAG | *rpoB* | antibiotic target replacement | Point mutations that occur in *M. leprae* *rpoB* result in resistance to RIF |
| aminoglycoside | 1_1370 | T | G | *rpsL* | antibiotic target alteration | Ribosomal protein S12 stabilizes the highly conserved |
| fusidic acid | 1_1372 | C | T | *fusA* | antibiotic target alteration | The mutations to this gene are involved in altering the translation elongation factor G (EF-G) in association with the ribosome to prevent fusidic acid from binding EF-G and preventing translation. |
| TET | 1_1400 | C/- | T | *rpsJ* | antibiotic target protection | *rpsJ* is a TET resistance protein identified in *Neisseria gonorrhoeae*. TET resistance is conferred by binding to the ribosome as a 30S ribosomal protection protein. |
| FQs | 1_1674 | - | CGT | *mfpA* | antibiotic target protection | *mfpA* is a qnr homolog, and a pentapeptide repeat protein that confers resistance to FQs in *M. smegmatis* |
| RIF | 1_2154 | A | C | *iri* | antibiotic inactivation | *iri* is a monooxygenase that confers resistance to RIF found in *Rhodothe coccus equi* |
| RIF, INH | 1_2439 | G | A | *efpA* | antibiotic efflux | *efpA* is an MFS transporter found in *M. tuberculosis.* |
| penam | 1_2480 | A | C | *blaF* | antibiotic inactivation | Class a beta-lactamase found in *M. fortuitum* |
| PAS | 1_2494 | C | T | *thyA* | antibiotic targets alteration | Point mutations in the thymidylate synthetase *thyA* it observes clinically to confer resistance to PAS. |
| PAS | 1_2610 | C | G | *ribD* | antibiotic target replacement | *ribD* is a riboflavin biosynthesis enzyme in *M. tuberculosis*. Point mutations in *ribD* cause enzyme overexpression, which allows the C-terminal reductase domain to act as an alternative dihydrofolate reductase. |
| INH, triclosan | 1_2838 | C | G | *inhA* | antibiotic target alteration | *inhA* is an enoyl-acyl carrier reductase used in lipid metabolism and fatty acid biosynthesis. It is inhibited by INH. Mutations in the promoter region or multiple copies of the *inhA* show marked resistance to INH-mediated inhibition of mycolic acid biosynthesis. |
| INH | 1_3096 | A | G | *katG* | antibiotic target alteration | *katG* is a catalase-peroxidase that catalyzes the activation of INH. INH inhibits mycolic acid synthesis, which prevents cell wall synthesis in mycobacteria |
| aminoglycoside | 1_3422 | T | G | *tlyA* | antibiotic target alteration | Specific mutations that arise in *M. tuberculosis* *tlyA*, resulting in aminoglycosides resistance |
| RIF | 1_3503 | G | A | *RbpA* | antibiotic target protection | RNA-polymerase binding protein which confers resistance to RIF |
| RIF, triclosan | 1_3781 | G | C | *kasA* | antibiotic target alteration | Specific mutations on the *M. tuberculosis* *kasA* result in the lowered affinity of INH, resulting in resistance |
| INH, triclosan | 1_3782 | C | A | *kasA* | antibiotic target alteration | Specific mutations on the *M. tuberculosis* *kasA* result in the lowered affinity of isoniazid, resulting in resistance |
| PAS | 1_4000 | T | C | *mupB* | antibiotic target alteration | *M. tuberculosis* *folC* with mutation conferring resistance to PAS |
| fosfomycin | 1_4331 | AA | - | *murA* | antibiotic target alteration | *M. tuberculosis murA* confers intrinsic resistance to fosfomycin. The presence of an aspartic acid residue in place of the critical cysteine at position 117 enables fosfomycin binding to be responsible for this intrinsic resistance. |
| TET | 1_4443 | C | G | *tap* | antibiotic efflux | Efflux pump proteins contained within mycobacterial genomes confer resistance to a number of different antibiotics including aminoglycosides, and TET. |
| polyamine | 1_4739 | G | C | *embR* | antibiotic target alteration | A point mutation in the *M. tuberculosis* *embR* results in increased resistance to ethambutol |
| RIF | 1_5083 | G | - | *arr-1* | antibiotic inactivation | *arr-1* is a chromosome-encoded ribosyltransferase found in *Mycobacterium smegmatis* |
| aminoglycoside | 1_5238 | GG | - | *APH(3'')-Ic* | antibiotic inactivation | APH(3'')-Ic is a chromosomal-encode aminoglycoside phosphotransferase in *M. fortuitum* |
| sulfonamide , sulfone | 1_5491 | T | G | *folP* | antibiotic target alteration | Dapsone inhibits bacterial synthesis of dihydrofolic acid by competing with para-aminobenzoate for the active site of dihydropteroate synthetase. |
| FQs | 1_5602 | - | T | *lrfA* | antibiotic efflux | *lfrA* is involved in the active efflux of quinolones and is found in *M. smegmatis*. |
| INH | 1_5821 | C | A | *katG* | antibiotic target alteration | *katG* is a catalase-peroxidase that catalyzes the activation of INH. INH inhibits mycolic acid synthesis, which prevents cell wall synthesis in mycobacteria. |
| polyamine | 1_5828 | A | G | *embC* | antibiotic target alteration | A point mutation in *M. tuberculosis* *embC* results in lower affinity between EMB and EmbC, as a result it shows resistance |
| polyamine | 1_5829 | C | T | *embA* | antibiotic target alteration | Specific mutations that occur in *M. tuberculosis* *embA* leaded lowered affinity of ethambutol to embA |
| polyamine | 1_5830 | T | G | *embB* | antibiotic target alteration | A point mutation that occurs within *M. bovis* *embB,* reveals the resistance to EMB |
| PZA | 1_5934 | A | G | *pncA* | antibiotic target alteration | *pncA* is a pyrazinamidase/nicotinamidase. It catalyzes the activation of PZA. Some mutations within *pncA* are associated with loss of enzyme activity, resulting in PZA resistance. |
| aminoglycoside | 1_6152 | T | C | *gidB* | antibiotic target alteration | Specific mutations that occur in *M. tuberculosis* *gidB* cause it to be streptomycin resistant |

Note: INH; isoniazid, RIF; rifampicin, FQs; fluoroquinolones, PZA; pyrazinamide, TET; tetracycline, PAS; para-aminosalicylic acid, STR: streptomycin. EMB: Ethambutol

Table S14 Predicted virulence genes analysis across the MF GZ001 and 21 other mycobacterial genomes.

Whereas, “P” indicates the presence of the predicted virulence gene.

“A” indicates the absence of the predicted virulence gene.

1. MF GZ001
2. *M*. *fortuitum* CT6
3. *M. abscessus* GZ002
4. *M. smegmatis* C^2^ 155
5. *M. tuberculosis*
6. *M. avium*
7. *M. canetti*
8. *M. chubuense*
9. *M. gilvum*
10. *M. intracellulare*
11. *M. kansasii*
12. *M. leprae*
13. *M. marinum*
14. *M. phlei*
15. *M. rhodesiae*
16. *M. ulcerans*
17. *M. vanbaalenii*
18. M. sp JLS
19. *M. sp* MCS
20. *M. africanum*
21. *M. mageritense*

| Genes | 1 | 2 | 3 | 4 | 5 | 6 | 7 | 8 | 9 | 10 | 11 | 12 | 13 | 14 | 15 | 16 | 17 | 18 | 19 | 20 | 21 |
| --- | --- | --- | --- | --- | --- | --- | --- | --- | --- | --- | --- | --- | --- | --- | --- | --- | --- | --- | --- | --- | --- |
| *glnA1* | P | P | P | P | P | P | P | P | P | P | P | P | P | P | P | P | P | P | P | P | P |
| *leuD* | P | P | P | P | P | P | P | P | P | P | P | P | P | P | P | P | P | P | P | P | P |
| *lysA* | P | P | P | P | P | P | P | P | P | P | P | P | P | P | P | P | P | P | P | P | P |
| *proC* | P | P | P | P | P | P | P | P | P | P | P | P | P | P | P | P | P | P | P | P | P |
| *purC* | P | P | P | P | P | P | P | P | P | P | P | P | P | P | P | P | P | P | P | P | P |
| *trpD* | P | A | P | A | A | A | A | A | A | A | A | P | A | A | A | P | A | A | A | A | A |
| *narX* | A | A | A | A | P | A | A | A | A | A | A | A | A | A | A | A | A | A | A | P | A |
| *narG* | P | P | A | A | P | P | P | P | P | P | P | A | A | A | P | A | P | P | P | P | P |
| *narH* | P | P | A | A | P | P | P | P | P | P | P | A | A | P | P | A | P | P | P | P | P |
| *narJ* | A | P | A | A | P | P | P | P | P | P | P | A | A | P | P | A | P | P | P | P | P |
| *narI* | P | P | A | A | P | P | P | P | P | P | P | A | A | P | P | A | P | P | P | P | P |
| *narK2* | P | P | P | P | P | A | P | P | P | A | P | A | A | P | P | A | P | P | P | P | P |
| *nuoG* | P | P | P | P | P | P | P | P | P | P | P | A | P | P | P | P | P | P | P | P | P |
| *erp* | A | P | P | P | P | P | P | P | P | P | A | P | P | P | P | P | P | P | P | P | A |
| *hbhA* | P | P | P | P | P | P | P | P | P | P | P | P | P | P | P | P | P | P | P | P | P |
| *mma4* | A | P | A | P | P | P | P | P | P | P | P | P | P | P | P | P | P | P | P | P | P |
| *cmaA2* | A | P | P | P | P | P | P | P | P | P | P | P | P | P | P | P | P | P | P | P | P |
| *fadD26* | A | P | A | P | P | A | P | P | P | A | P | P | P | A | A | P | P | P | P | P | A |
| *fadD28* | P | P | P | P | P | P | P | P | P | P | P | P | P | A | A | P | P | P | P | P | P |
| *ppsA* | A | A | A | P | P | A | P | P | P | A | P | P | P | A | A | P | P | P | P | P | A |
| *ppsB* | A | A | A | P | P | A | P | P | P | A | P | P | P | A | A | P | P | P | P | P | A |
| *ppsC* | A | A | A | A | P | A | P | A | A | A | P | P | P | A | A | P | A | P | P | P | A |
| *ppsD* | A | A | A | P | P | A | P | P | P | A | P | P | P | A | A | P | P | A | A | P | A |
| *ppsE* | A | A | A | P | P | A | P | P | P | A | P | P | P | A | A | P | P | P | P | P | A |
| *papA5* | A | A | A | A | P | A | P | P | P | A | P | P | P | A | A | P | P | P | P | P | A |
| *mas* | A | P | P | A | P | P | P | A | A | P | P | P | P | P | A | P | A | P | P | P | P |
| *mmpL7* | A | A | A | A | P | A | P | A | A | A | P | P | P | A | A | P | A | A | A | P | A |
| *ddrA* | A | A | A | P | P | P | P | P | P | P | P | P | P | P | P | P | P | P | P | P | A |
| *ddrB* | A | A | A | P | P | P | P | P | P | P | P | A | P | A | P | P | P | P | P | P | A |
| *drrC* | A | A | A | A | P | P | P | P | P | P | P | P | P | A | P | P | P | P | P | P | A |
| *tesA* | A | A | A | A | A | A | A | A | A | A | A | A | A | A | A | A | A | A | A | A | A |
| *lppx* | A | A | A | A | A | A | A | A | A | A | A | A | A | A | A | A | A | A | A | A | A |
| *pks15* | P | A | A | A | A | A | A | A | A | A | A | A | A | A | A | A | A | A | A | A | A |
| *pks1* | P | A | A | A | A | A | A | A | A | A | A | A | A | A | A | A | A | A | A | A | A |
| *pks15P1* | P | A | A | A | A | A | A | A | A | A | A | A | A | A | A | A | A | A | A | A | A |
| *fadD22* | A | A | A | A | A | A | A | A | A | A | A | A | A | A | A | A | A | A | A | A | A |
| *fadD29* | A | A | A | A | A | A | A | A | A | A | A | A | A | A | A | A | A | A | A | A | A |
| *pcaA* | P | P | P | P | P | P | P | P | P | P | P | P | P | P | P | P | P | P | P | P | P |
| *stf0* | P | P | P | P | P | P | P | A | A | P | P | A | P | P | P | A | A | P | P | P | P |
| *papA2* | A | P | P | P | P | P | P | P | P | P | A | A | A | P | A | A | P | P | P | P | P |
| *papA1* | A | A | A | A | P | A | P | A | A | A | A | A | A | P | A | A | A | P | P | P | P |
| *pks2* | A | P | P | A | P | P | P | A | A | P | P | P | P | P | A | P | A | P | P | P | P |
| *mmpL8* | A | A | A | A | P | A | P | A | A | A | A | A | A | P | A | A | A | P | P | P | P |
| *kasB* | p | P | P | P | P | P | P | P | P | P | P | P | P | P | P | P | P | P | P | P | P |
| *icl* | P | P | P | P | P | P | P | P | P | P | P | A | P | P | P | P | P | P | P | P | P |
| *lipF* | A | P | A | P | P | P | P | P | P | P | P | P | P | P | P | P | P | P | P | P | P |
| *sapM* | A | A | A | P | P | P | P | P | A | P | P | A | P | A | A | P | A | A | A | P | A |
| *panC* | P | P | P | P | P | P | P | P | P | P | P | P | P | P | P | P | P | P | P | P | P |
| *panD* | P | P | P | P | P | P | P | P | P | P | P | P | P | P | P | P | P | P | P | P | P |
| *plcA* | A | A | A | A | P | A | P | A | A | A | P | A | P | A | A | P | A | A | A | P | A |
| *plcB* | A | A | A | A | P | A | P | A | A | A | P | A | P | A | A | P | A | A | A | P | A |
| *plcC* | A | A | A | A | P | A | P | A | A | A | P | A | P | A | A | P | A | A | A | P | A |
| *plcD* | A | A | A | A | P | A | P | A | A | A | P | A | P | A | A | P | A | A | A | P | A |
| *mce1A* | P | P | A | P | P | P | P | P | P | P | P | P | P | P | P | P | P | P | P | P | P |
| *mce1B* | P | P | A | P | P | P | P | P | P | P | P | P | P | P | P | P | P | P | P | P | P |
| *mce1C* | P | P | A | P | P | P | P | P | P | P | A | P | P | P | P | P | P | P | P | P | P |
| *mce1D* | P | P | A | P | P | P | P | P | P | P | P | P | P | P | P | P | P | P | P | P | P |
| *mce1E* | P | P | A | P | P | P | P | P | P | P | P | P | P | P | P | P | P | P | P | P | P |
| *mce1F* | P | P | A | P | P | P | P | P | P | P | P | P | P | P | P | P | P | P | P | P | P |
| *mce2A* | A | P | A | P | P | P | P | P | P | P | P | P | P | P | P | P | P | P | P | P | P |
| *mce2B* | A | P | A | P | P | P | P | P | P | P | P | P | P | P | P | P | P | P | P | P | P |
| *mce2C* | A | P | A | P | P | P | P | P | P | P | A | P | P | P | P | P | P | P | P | P | P |
| *mce2D* | A | P | A | P | P | P | P | P | P | P | P | P | P | P | P | P | P | P | P | P | P |
| *mce2E* | A | P | A | P | P | P | P | P | P | P | P | P | P | P | P | P | P | P | P | P | P |
| *mce2F* | A | P | A | P | P | P | P | P | P | P | P | P | P | P | P | P | P | P | P | P | P |
| *mce3A* | A | P | A | P | P | P | P | P | P | P | P | A | P | P | P | P | P | P | P | A | A |
| *mce3B* | A | P | A | P | P | P | P | P | P | P | P | A | P | P | P | P | P | P | P | A | A |
| *mce3C* | A | P | A | P | P | P | P | P | P | P | A | A | P | P | P | P | P | P | P | A | A |
| *mce3D* | P | P | A | P | P | P | P | P | P | P | P | A | P | P | P | P | P | P | P | A | A |
| *mce3E* | P | P | A | P | P | P | P | P | P | P | P | A | P | P | P | P | P | P | P | A | A |
| *mce3F* | P | P | A | P | P | P | P | P | P | P | P | A | P | P | P | P | P | P | P | A | A |
| *mce4A* | P | P | P | P | P | P | P | P | P | P | P | A | P | P | P | P | P | P | P | P | P |
| *mce4B* | P | P | P | P | P | P | P | P | P | P | P | A | P | P | P | P | P | P | P | P | P |
| *mce4C* | P | P | P | P | P | P | P | P | P | P | P | A | P | P | P | P | P | P | P | P | P |
| *mce4D* | P | P | P | P | P | P | P | P | P | P | P | A | P | P | P | P | P | P | P | P | P |
| *mce4E* | P | P | P | P | P | P | P | P | P | P | P | A | P | P | P | P | P | P | P | P | P |
| *mce4F* | P | P | P | P | P | P | P | P | P | P | P | A | P | P | P | P | P | P | P | P | A |
| *mce5A* | P | P | P | A | A | P | A | A | P | P | A | A | P | P | A | P | P | A | A | A | P |
| *mce5B* | P | P | P | A | A | P | A | A | P | P | A | A | P | P | A | P | P | A | A | A | P |
| *mce5C* | P | P | P | A | A | P | A | A | P | P | A | A | P | P | A | P | P | A | A | A | P |
| *mce5D* | P | P | P | A | A | P | A | A | P | P | A | A | P | P | A | P | P | A | A | A | P |
| *mce5E* | P | P | P | A | A | P | A | A | P | P | A | A | P | P | A | P | P | A | A | A | P |
| *mce5F* | P | P | P | A | A | P | A | A | P | P | A | A | P | P | A | P | P | A | A | A | P |
| *mce6A* | A | P | P | A | A | P | A | A | A | P | A | A | P | P | A | P | P | A | A | A | P |
| *mce6B* | A | P | P | A | A | P | A | A | A | P | A | A | P | A | A | P | P | A | A | A | P |
| *mce6C* | A | P | P | A | A | A | A | A | A | A | A | A | P | A | A | P | A | A | A | A | P |
| *mce6D* | P | P | P | A | A | P | A | A | P | P | A | A | P | P | A | P | P | A | A | A | P |
| *mce6E* | A | P | P | A | A | P | A | A | P | P | A | A | P | A | A | P | P | A | A | A | P |
| *mce6F* | P | P | P | A | A | P | A | A | P | P | A | A | P | P | A | P | P | A | A | A | P |
| *mce7A* | P | P | A | P | A | P | A | P | P | P | P | A | P | P | P | A | P | P | P | A | P |
| *mce7B* | P | P | A | P | A | P | A | P | P | P | P | A | P | P | P | A | P | P | P | A | P |
| *mce7C* | P | P | A | P | A | P | A | P | P | P | P | A | P | P | P | A | P | P | P | A | P |
| *mce7D* | P | P | A | P | A | P | A | P | P | P | P | A | P | P | P | A | P | P | P | A | P |
| *mce7E* | P | P | A | P | A | P | A | P | P | P | A | A | P | P | P | A | P | P | P | A | P |
| *mce7F* | P | P | A | P | A | P | A | P | P | P | P | A | P | P | P | A | P | P | P | A | P |
| *mce8A* | P | P | A | P | A | P | A | P | P | P | P | A | P | P | A | A | P | P | P | A | P |
| *mce8B* | P | P | A | P | A | P | A | P | P | P | P | A | P | P | P | A | P | P | P | A | P |
| *mce8C* | P | P | A | P | A | P | A | P | P | P | P | A | P | P | P | A | P | P | P | A | P |
| *mce8D* | A | P | A | P | A | P | A | P | P | P | P | A | P | P | P | A | P | P | P | A | P |
| *mce8E* | A | P | A | P | A | P | A | P | P | P | A | A | P | P | P | A | P | P | P | A | P |
| *mce8F* | A | P | A | P | A | P | A | P | P | P | P | A | P | P | P | A | P | P | P | A | P |
| *mce9A* | A | P | P | A | A | P | A | A | P | P | A | A | P | P | A | P | P | A | A | A | P |
| *mce9B* | A | P | P | A | A | P | A | A | P | P | A | A | P | P | A | P | P | A | A | A | P |
| *mce9C* | A | P | P | A | A | P | A | A | P | P | A | A | P | P | A | P | P | A | A | A | P |
| *mce9D* | A | P | P | A | A | P | A | A | P | P | A | A | P | P | A | P | P | A | A | A | P |
| *mce9E* | A | P | P | A | A | P | A | A | P | P | A | A | P | P | A | P | P | A | A | A | P |
| *mce9F* | A | P | P | A | A | P | A | A | P | P | A | A | P | P | A | P | P | A | A | A | P |
| *irtA* | P | P | P | A | P | P | P | A | A | P | A | A | P | A | P | P | A | A | A | P | P |
| *irtB* | P | P | P | A | P | P | P | A | A | P | A | A | P | A | P | P | A | A | A | P | P |
| *fxuC* | A | A | A | A | A | A | A | A | A | A | A | A | A | A | A | A | A | A | A | A | A |
| *fxuA* | A | A | A | A | A | A | A | A | A | A | A | A | A | A | A | A | A | A | A | A | A |
| *fxuB* | A | A | A | A | A | A | A | A | A | A | A | A | A | A | A | A | A | A | A | A | A |
| *fxbA* | A | A | A | A | A | A | A | A | A | A | A | A | A | A | A | A | A | A | A | A | A |
| *exiT* | A | A | A | A | A | A | A | A | A | A | A | A | A | A | A | A | A | A | A | A | A |
| *fxbBC* | A | A | A | A | A | A | A | A | A | A | A | A | A | A | A | A | A | A | A | A | A |
| *fxuD* | A | A | A | A | A | A | A | A | A | A | A | A | A | A | A | A | A | A | A | A | A |
| *ideR* | P | P | P | P | P | P | P | P | P | P | P | P | P | P | P | P | P | P | P | P | P |
| *mgtC* | A | A | P | A | P | P | P | A | A | P | P | A | P | A | A | P | A | P | P | P | A |
| *mbtH* | P | P | P | P | P | P | P | P | P | P | P | A | P | P | P | P | P | P | P | P | P |
| *mbtG* | p | P | P | A | P | P | P | A | P | P | P | A | P | P | P | P | P | P | P | P | A |
| *mbtF* | P | P | P | A | P | P | P | A | P | P | P | A | P | P | P | P | P | P | P | P | P |
| *mbtE* | P | P | P | A | P | P | P | A | P | P | P | A | P | P | P | P | P | P | P | P | P |
| *mbtD* | P | P | P | A | P | P | P | A | P | P | P | A | P | A | A | P | P | P | P | P | P |
| *mbtC* | P | P | P | A | P | P | P | A | P | P | P | A | P | P | P | P | P | P | P | P | A |
| *mbtB* | P | P | P | A | P | P | P | A | P | P | P | A | P | P | P | P | P | P | P | P | P |
| *mbtA* | P | P | P | A | P | P | P | A | P | P | P | A | P | P | P | P | P | P | P | P | P |
| *mbtJ* | A | P | P | P | P | P | P | P | A | P | P | A | P | A | P | P | A | A | A | P | P |
| *mbtI* | A | P | P | A | P | P | P | A | A | P | P | A | P | A | A | P | A | A | A | P | P |
| *fadD33* | P | P | A | A | P | P | P | A | P | P | A | A | A | P | A | A | P | P | P | P | P |
| *fadE14* | P | P | A | A | P | P | P | A | A | P | A | A | A | P | A | A | P | P | P | P | A |
| *relA* | P | P | P | P | P | P | P | P | P | P | P | P | P | P | P | P | P | P | P | P | P |
| *devRPdosR* | P | P | P | A | P | P | P | P | P | A | P | A | P | P | P | P | P | P | P | P | P |
| *devS* | A | P | P | A | P | P | P | P | P | A | P | A | P | P | P | P | P | P | P | P | P |
| *mprA* | P | P | P | P | P | P | P | P | P | P | P | P | P | P | P | P | P | P | P | P | P |
| *mprB* | P | P | P | P | P | P | P | P | P | P | P | P | P | P | P | P | P | P | P | P | P |
| *phoP* | P | P | P | P | P | P | P | P | P | P | P | A | P | P | P | P | P | P | P | P | P |
| *phoR* | P | P | P | P | P | P | P | P | P | P | P | A | P | P | P | P | P | P | P | P | P |
| *prrA* | P | P | P | P | P | P | P | P | P | P | P | P | P | P | P | P | P | P | P | P | P |
| *prrB* | P | P | P | P | P | P | P | P | P | P | P | P | P | P | P | P | P | P | P | P | P |
| *SigAP/rpoV* | P | P | P | P | P | P | P | P | P | P | P | P | P | P | P | P | P | P | P | P | A |
| *sigE* | P | P | P | P | P | P | P | P | P | P | P | P | P | P | P | P | P | P | P | P | P |
| *sigF* | P | P | P | P | P | P | P | P | P | P | P | A | P | P | P | P | P | P | P | P | P |
| *sigH* | P | P | P | P | P | P | P | P | P | P | P | A | P | P | P | P | P | P | P | P | P |
| *sigM* | P | P | P | P | P | P | P | P | P | P | P | A | P | P | P | P | P | P | P | P | P |
| *whiB3* | P | P | P | P | A | P | A | P | P | P | P | P | P | P | P | P | P | A | A | P | A |
| *lpqH* | A | P | P | P | P | P | P | P | P | P | P | P | P | P | P | P | P | P | P | P | P |
| *hspX* | A | A | P | A | P | A | P | P | A | P | P | A | P | P | P | A | P | P | P | P | A |
| *fbpA* | P | P | P | P | P | P | P | P | P | P | P | P | P | P | P | P | P | P | P | P | P |
| *fbpB* | P | P | P | P | P | P | P | P | P | P | P | P | P | P | P | P | P | P | P | P | P |
| *fbpC* | P | P | P | P | P | P | P | P | P | P | P | P | P | P | P | P | P | P | P | P | P |
| *eis* | A | P | P | A | P | A | P | A | P | A | P | A | P | P | A | A | P | P | P | P | P |
| *pknG* | P | P | P | P | P | P | P | P | P | P | P | P | P | P | P | P | P | P | P | P | P |
| *secA2* | P | P | P | P | P | P | P | P | P | P | P | P | P | P | P | P | P | P | P | P | P |
| *esxA* | P | P | A | A | P | A | P | A | P | A | P | P | P | P | P | A | P | P | P | P | P |
| *esxB* | P | P | A | A | P | A | P | A | P | A | P | A | P | P | P | A | P | P | P | P | P |
| *eccA1* | P | P | A | A | P | A | P | A | P | A | P | P | P | P | P | A | P | P | P | P | P |
| *eccB1* | P | P | A | A | P | A | P | A | P | A | P | P | P | P | P | A | P | P | P | P | A |
| *eccCa1* | P | P | A | A | P | A | P | A | P | A | P | P | P | P | P | A | P | P | P | P | P |
| *eccCb1* | P | P | A | A | P | A | P | A | P | A | P | P | P | P | P | A | P | P | P | P | P |
| *PE35* | A | P | A | A | P | A | P | A | P | A | P | A | P | P | P | A | P | P | P | P | P |
| *eccD1* | P | P | A | A | P | A | P | P | P | A | P | P | P | P | P | P | P | P | P | P | P |
| *espK* | A | A | A | A | A | A | A | A | A | A | P | A | P | A | A | A | A | A | A | A | A |
| *eccE1* | P | P | A | A | P | A | P | P | P | A | P | P | P | P | P | A | P | P | P | P | P |
| *mycP1* | P | P | A | A | P | A | P | P | P | A | P | P | P | P | P | A | P | P | P | P | P |
| *espD* | A | A | A | A | P | A | P | A | A | A | A | P | P | A | A | A | A | A | A | P | A |
| *espC* | A | A | A | A | P | A | P | A | A | A | P | P | P | A | A | A | A | A | A | P | A |
| *espA* | A | A | A | A | P | A | P | A | A | A | P | P | P | A | A | A | A | A | A | P | A |
| *espB* | A | A | A | A | P | A | P | A | P | A | P | A | P | A | A | A | P | P | P | P | A |
| *PPE68* | P | P | A | A | P | A | P | A | P | A | P | P | P | P | P | A | P | P | P | P | P |
| *espI* | P | P | P | P | P | P | P | P | P | P | P | P | P | P | P | P | P | P | P | P | P |
| *espJ* | A | A | A | A | P | A | P | A | P | A | P | A | P | A | A | P | P | A | A | P | A |
| *espL* | A | P | A | A | P | A | P | P | P | A | P | P | P | P | P | P | P | P | P | P | P |
| *espR* | P | P | P | P | P | P | P | P | P | P | P | P | P | P | P | P | P | P | P | P | P |
| *eccA2* | A | A | A | A | P | P | P | A | A | P | P | A | A | A | A | A | A | A | A | P | A |
| *eccE2* | A | A | A | A | P | P | P | A | A | P | P | A | A | A | A | A | A | A | A | P | A |
| *mycP2* | A | A | A | A | P | P | P | A | A | P | P | A | A | A | A | A | A | A | A | P | A |
| *eccD2* | A | A | A | A | P | P | P | A | A | P | P | A | A | A | A | A | A | A | A | P | A |
| *espG2* | A | A | A | A | P | P | P | A | A | P | P | A | A | A | A | A | A | A | A | P | A |
| *esxC* | A | A | A | A | P | P | P | A | A | P | P | A | A | A | A | A | A | A | A | A | A |
| *esxD* | A | A | A | A | P | P | P | A | A | P | P | A | A | A | A | A | A | A | A | P | A |
| *PPE69* | P | A | A | A | P | P | P | A | A | P | P | A | A | A | A | A | A | A | A | P | A |
| *PE36* | P | A | A | A | P | P | P | A | A | P | P | A | A | A | A | A | A | A | A | P | A |
| *eccC2* | A | A | A | A | P | P | P | A | A | P | P | A | A | A | A | A | A | A | A | P | A |
| *eccB2* | A | A | A | A | P | P | P | A | A | P | P | A | A | A | A | A | A | A | A | P | A |
| *eccA3* | P | P | P | A | P | P | P | A | P | P | P | P | P | P | P | P | P | P | P | P | P |
| *eccB3* | P | P | P | A | P | P | P | A | P | P | P | P | P | P | P | P | P | P | P | P | P |
| *eccC3* | P | P | P | A | P | P | P | A | P | P | P | P | P | P | P | P | P | P | P | P | A |
| *PE5* | A | P | P | A | P | P | P | A | P | P | P | P | P | P | P | P | P | P | P | P | P |
| *PPE4* | A | A | P | A | P | P | P | A | P | P | A | A | P | P | A | P | P | P | P | P | A |
| *esxH* | P | P | P | A | P | P | P | A | P | P | P | P | P | P | P | P | P | P | P | P | P |
| *espG3* | P | P | P | A | P | P | P | A | P | P | P | P | P | P | P | P | P | P | P | P | P |
| *eccD3* | P | P | P | A | P | P | P | A | P | P | P | P | P | P | P | P | P | P | P | P | P |
| *mycP3* | P | P | P | A | P | P | P | A | P | P | P | P | P | P | P | P | P | P | P | P | P |
| *eccE3* | P | P | P | A | P | P | P | A | P | P | P | P | P | A | A | P | P | P | P | P | P |
| *esxG* | P | P | P | A | P | P | P | A | P | P | P | P | P | P | P | P | P | P | P | P | P |
| *esxT* | A | P | A | A | P | P | P | P | P | P | P | A | P | P | P | P | P | P | P | P | P |
| *esxU* | A | P | A | A | P | P | P | P | P | P | P | A | P | P | P | P | P | P | P | P | P |
| *eccC4* | A | P | P | A | P | P | P | P | P | P | P | A | P | A | A | P | P | P | P | P | P |
| *cccD4* | A | A | P | A | P | P | P | A | A | A | A | A | P | A | A | P | P | P | P | P | A |
| *mycP4* | A | P | P | A | P | P | P | P | P | P | P | A | A | P | P | A | P | P | P | P | P |
| *eccB4* | A | A | P | A | P | P | P | P | P | P | P | A | P | A | A | P | P | P | P | P | A |
| *eccA5* | A | A | A | A | P | P | P | A | A | P | P | P | P | A | A | P | A | A | A | P | A |
| *eccE5* | A | A | A | A | P | P | P | A | A | P | P | P | P | A | A | P | A | A | A | P | A |
| *mycP5* | A | A | A | A | P | P | P | A | A | P | P | P | P | A | A | P | A | A | A | P | A |
| *eccD5* | A | A | A | A | P | P | P | A | A | P | P | P | P | A | A | P | A | A | A | P | A |
| *esxN* | A | A | A | A | P | P | P | A | A | P | P | P | P | A | A | P | A | A | A | P | A |
| *esxM* | A | A | A | A | P | P | P | A | A | P | P | P | P | A | A | P | A | A | A | P | A |
| *eccCb5* | A | A | A | A | P | P | P | A | A | P | P | P | P | A | A | P | A | A | A | P | A |
| *eccCa5* | A | A | A | A | P | P | P | A | A | P | P | P | P | A | A | P | A | A | A | P | A |
| *eccB5* | A | A | A | A | P | P | P | A | A | P | P | P | P | A | A | P | A | A | A | P | A |
| *PPE41* | A | A | A | A | P | A | P | A | A | A | A | A | A | A | A | A | A | A | A | P | A |
| *PPE25* | A | A | A | A | P | P | P | A | A | P | P | A | P | A | A | P | A | A | A | P | A |
| *PE18* | A | A | A | A | P | P | P | A | A | P | P | A | P | A | A | P | A | A | A | P | A |
| *PPE26* | A | A | A | A | P | P | P | A | A | P | P | A | P | A | A | P | A | A | A | P | A |
| *PPE27* | A | A | A | A | P | A | P | A | A | A | P | A | A | A | A | A | A | A | A | P | A |
| *PE19* | A | A | A | A | P | P | P | A | A | P | P | A | P | A | A | P | A | A | A | P | A |
| *ahpC* | A | A | P | A | P | P | P | A | A | P | P | P | P | A | A | P | A | A | A | P | A |
| *katG* | P | P | P | P | P | P | P | P | P | P | P | A | P | P | P | P | P | P | P | P | P |
| *sodC* | P | P | P | P | P | P | P | P | P | P | P | P | P | P | P | P | P | P | P | P | P |
| *sodA* | P | P | P | A | P | P | P | A | A | P | P | P | P | A | A | P | A | P | P | P | P |
| *mlsA1* | A | A | A | A | A | A | A | A | A | A | A | A | A | A | A | A | A | A | A | A | A |
| *mlsA2* | A | A | A | A | A | A | A | A | A | A | A | A | A | A | A | A | A | A | A | A | A |
| *mlsB* | A | A | A | A | A | A | A | A | A | A | A | A | A | A | A | A | A | A | A | A | A |
| *rmlA* | P | A | A | P | A |  | A | A | A | A | A | A | A | A | A | A | A | A | A | A | A |
| *mmpL4b* | P | A | A | A | A | P | A | A | A | A | A | A | A | A | A | A | A | A | A | A | A |
| *zmp1* | P | A | A | P | A | A | A | A | A | A | A | A | A | A | A | A | A | A | A | A | A |
| *mmpL11* | P | A | A | P | A | A | A | A | A | A | A | A | A | A | A | A | A | A | A | A | A |
| *Rv0204c* | P | A | A | A | P | A | A | A | A | A | A | A | A | A | A | A | P | A | A | A | A |
| *mmpL3* | P | A | A | A | A | A | A | A | A | A | A | A | A | A | A | A | A | A | A | A | A |
| *fadE5* | P | A | A | P | A | A | A | A | A | A | A | A | A | A | A | A | A | A | A | A | A |
| *Rv0926* | P | A | A | A | P | A | A | A | A | A | A | A | A | A | A | A | A | A | A | A | A |
| *papA3* | P | A | A | P | A | A | A | A | A | A | A | A | A | A | A | A | A | A | A | A | A |
| *mmpL10* | P | A | A | A | A | A | A | A | A | A | A | A | A | A | A | A | P | A | A | A | A |
| *PE* | P | A | P | A | A | A | A | A | A | A | A | A | A | A | A | A | A | A | A | A | A |
| *fad23* | P | A | A | A | A | A | A | A | P | A | A | A | A | A | A | A | A | A | A | A | A |
| *mmpL4a* | P | A | A | P | A | A | A | A | A | A | A | A | A | A | A | A | A | A | A | A | A |
| *sigL* | P | A | A | P | A | A | A | A | A | A | A | A | A | A | A | A | A | A | A | A | A |
| *Rv0440* | P | A | A | A | P | A | A | A | A | A | A | A | A | A | A | A | A | A | A | A | A |
| *mbtK* | P | A | A | P | A | A | A | A | A | A | A | A | A | A | A | A | A | A | A | A | A |
| *mps1* | P | A | A | A | P | A | A | A | A | A | A | A | A | A | A | A | A | A | A | A | A |
| *ecf* | P | A | A | A | A | A | A | A | A | A | A | A | A | A | A | A | A | P | A | A | A |
| *cyp125* | P | A | A | P | A | A | A | A | A | A | A | A | A | A | A | A | A | P | A | A | A |
| *Rv1837c* | P | A | A | A | P | A | A | A | A | A | A | A | A | A | A | A | A |  | A | A | A |
| *icl2* | P | A | A | A | A | A | A | A | A | A | A | A | A | A | A | A | P | A | A | A | A |
| *pafA* | P | A | A | P | A | A | A | A | A | A | A | A | A | A | A | A | A | A | A | A | A |
| *mpa* | P | A | A | A | A | P | A | A | A | A | A | A | A | A | A | A | A | A | A | A | A |
| *adhD* | P | A | A | A | A | A | A | A | A | P | A | A | A | A | A | A | P | A | A | A | A |
| *caeA* | P | A | A | A | A | A | A | A | A | A | A | A | A | A | A | A | A | A | A | A | A |
| *ptpA* | P | A | A | A | A | A |  | A | A | A | A | A | A | A | A | A | A | P | A | A | A |
| *sadH* | P | A | A | A | A | A | P | A | A | A | A | A | A | A | A | A | A | A | A | A | A |
| *sugC* | P | A | A | P | A | A | A | A | A | A | A | A | A | A | A | A | A | A | A | A | A |
| *sugB* | P | A | A | P | A | A | A | A | A | A | A | A | A | A | A | A | A | A | A | A | A |
| *sugA* | P | A | A | A | A | A | A | A | A | A | A | A | A | A | A | A | A | P | A | A | A |
| *lpqY* | P | A | A | P | A | A | A | A | A | A | A | A | A | A | A | A | A | A | A | A | A |
| *regX3* | P | A | A | A | A | A | A | A | A | A | A | A | A | A | A | A | A | P | A | A | A |
| *senX3* | P | A | A | A | A | A | A | A | A | A | A | A | A | A | A | A | A | A | A | A | A |
| *fadE29* | P | A | A | A | A | A | A | A | A | A | A | A | A | A | A | A | P | A | A | A | A |
| *fadE28* | P | A | A | P | A | A | A | A | A | A | A | A | A | A | A | A | A | A | A | A | A |
| *ctpC* | P | A | A | P | A | A | A | A | A | A | A | A | A | A | A | A | A | A | A | A | A |
