# Supplementary figures and images for: Comparative genome analysis reveals high-level drug resistance markers in a clinical isolate of Mycobacterium fortuitum subsp. fortuitum MF GZ001
Source: Front Cell Infect Microbiol. 2023 Jan 4;12:1056007. doi: 10.3389/fcimb.2022.1056007 (PMC9846761; doi:10.3389/fcimb.2022.1056007)

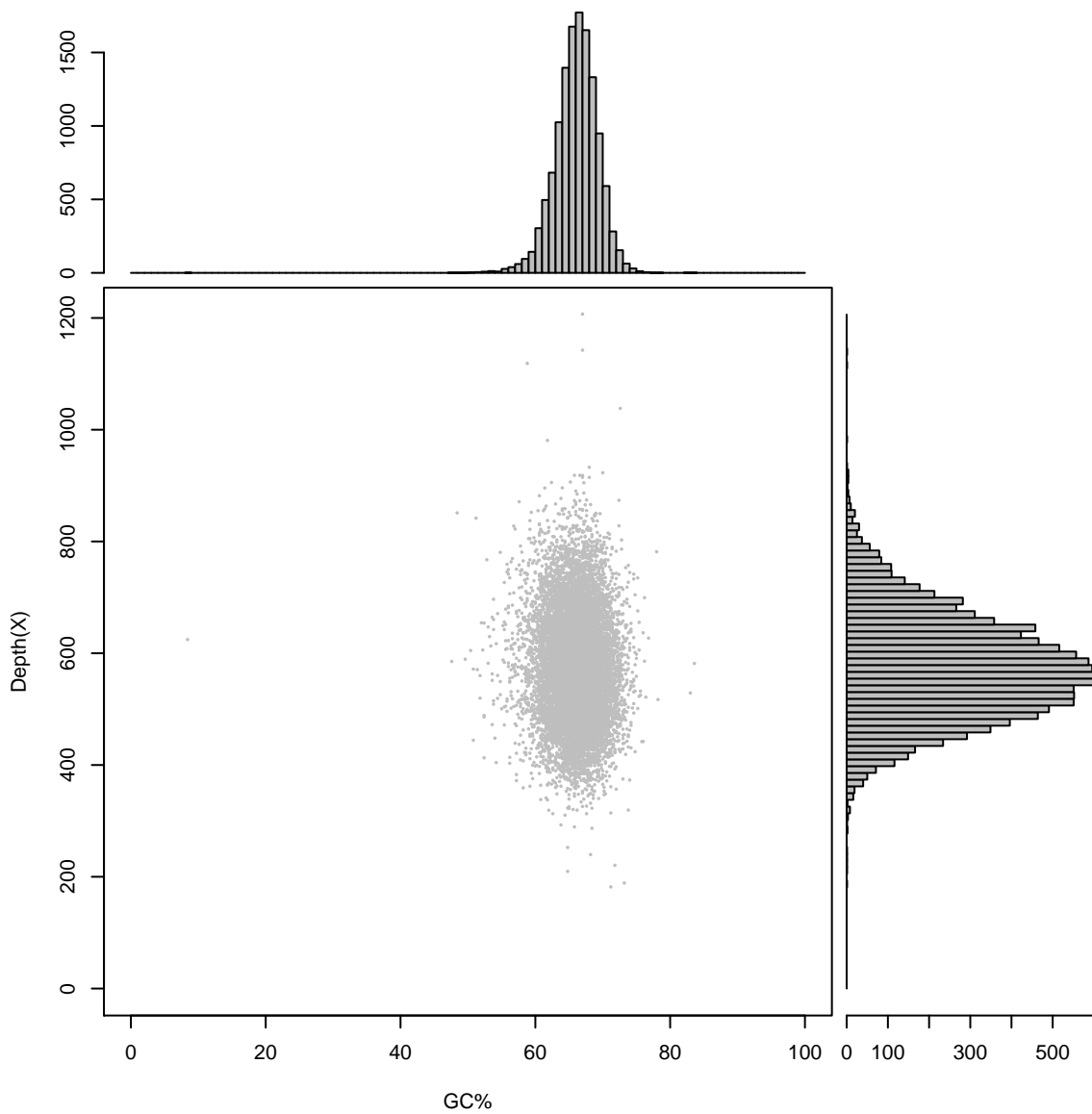

Supplement: Supplementary file 1 [file DataSheet_1.zip › Figure S1 .pdf]

Pathway categories

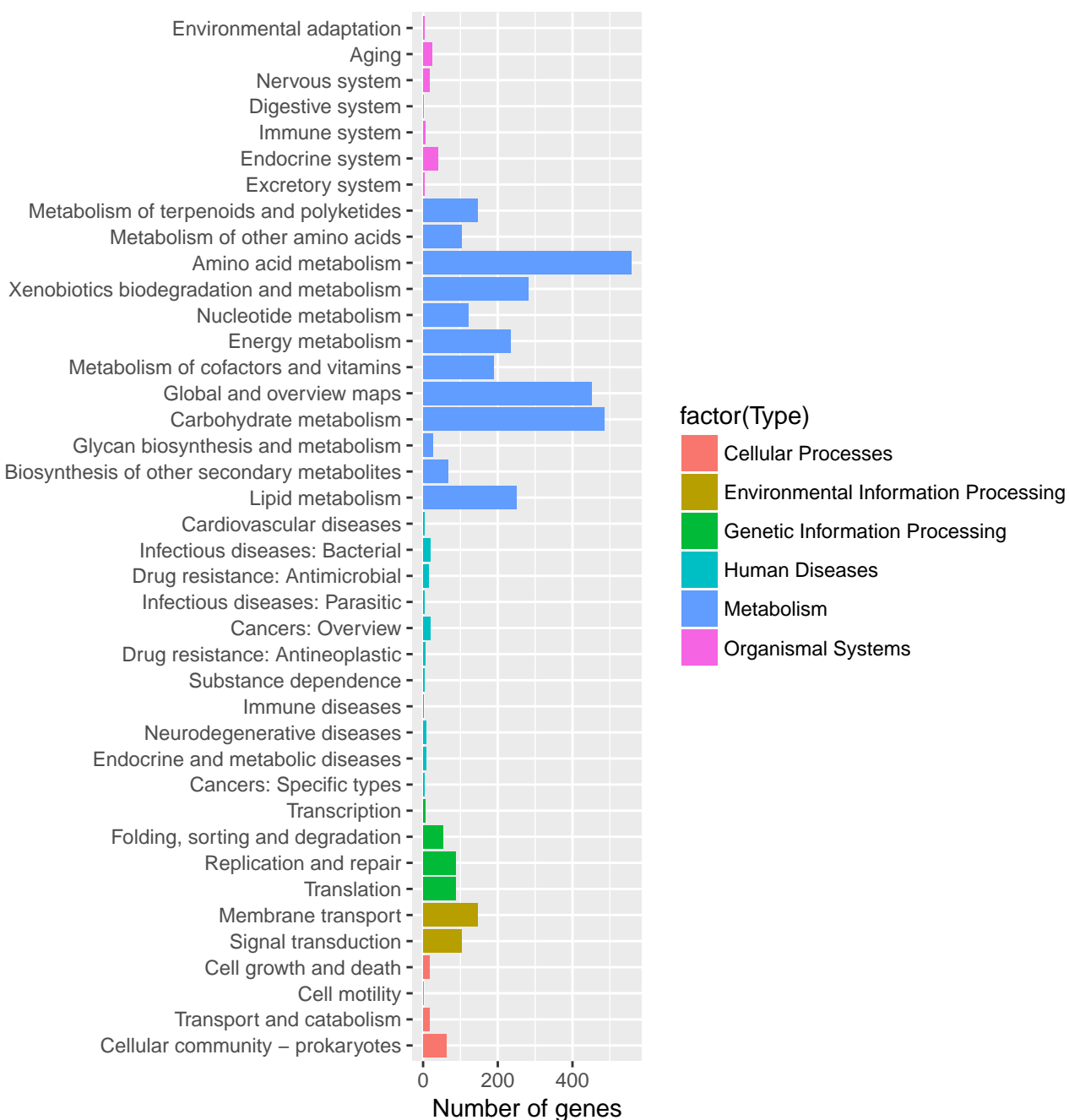

Supplement: Supplementary file 1 [file DataSheet_1.zip › Figure S2.pdf]

### NR homologous species distribution

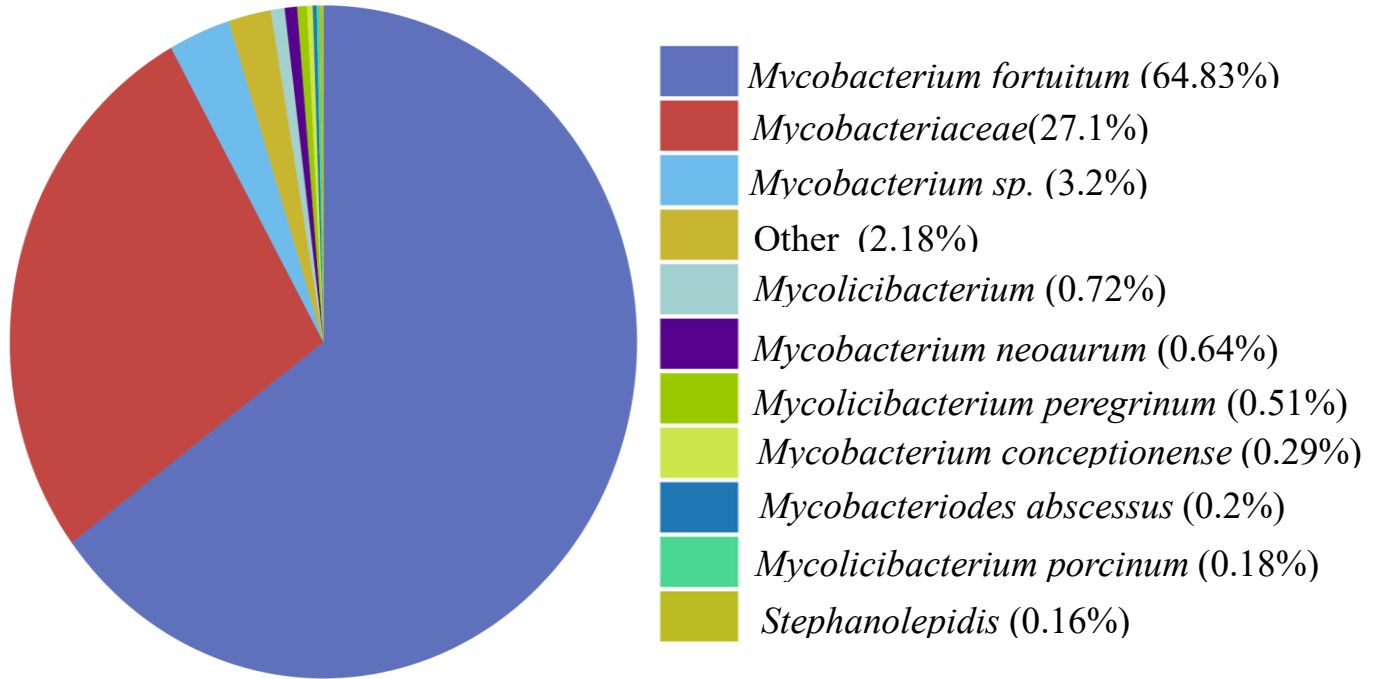

Supplement: Supplementary file 1 [file DataSheet_1.zip › Figure S4.pdf]

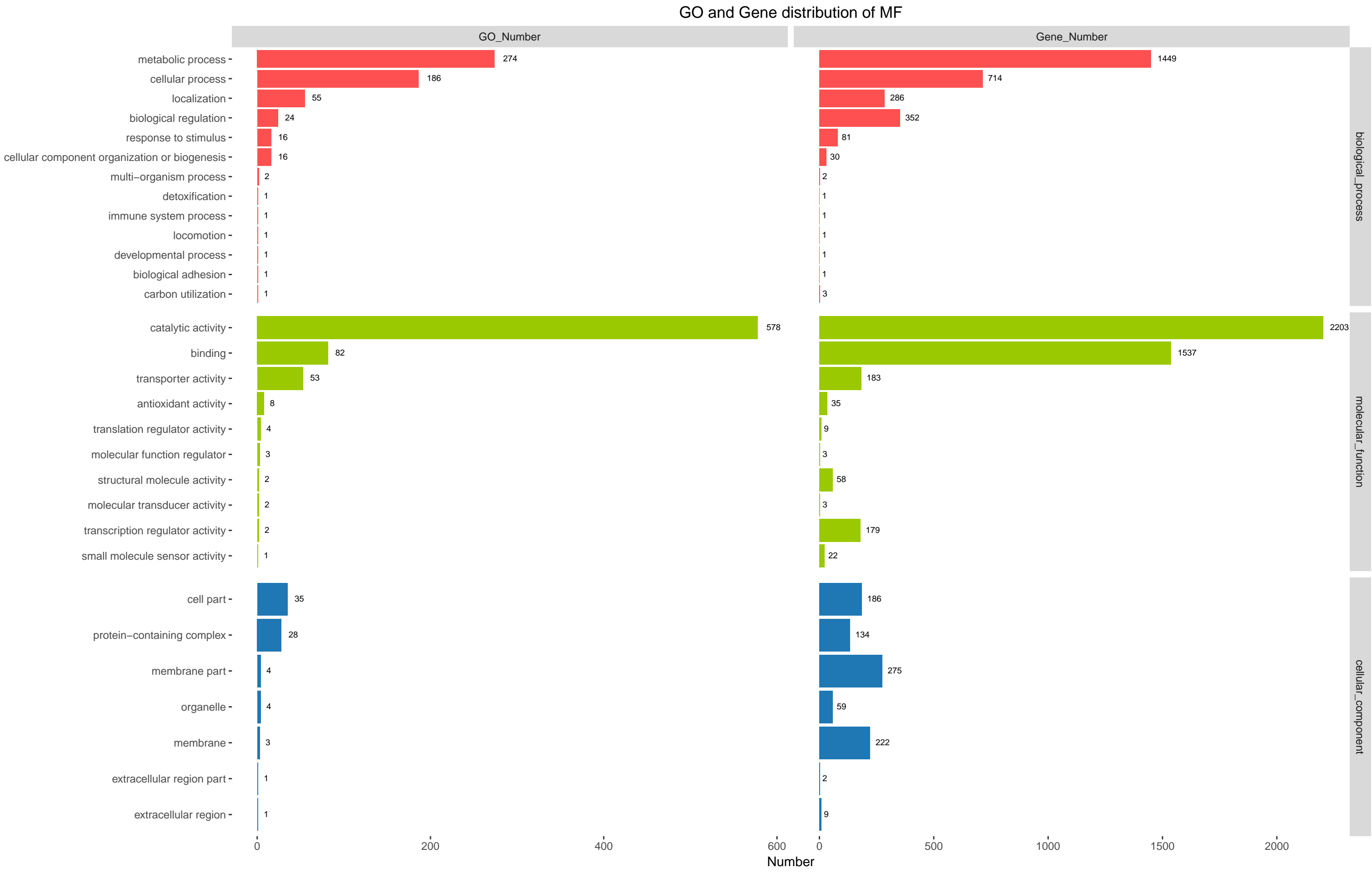

Supplement: Supplementary file 1 [file DataSheet_1.zip › Figure S5.pdf]

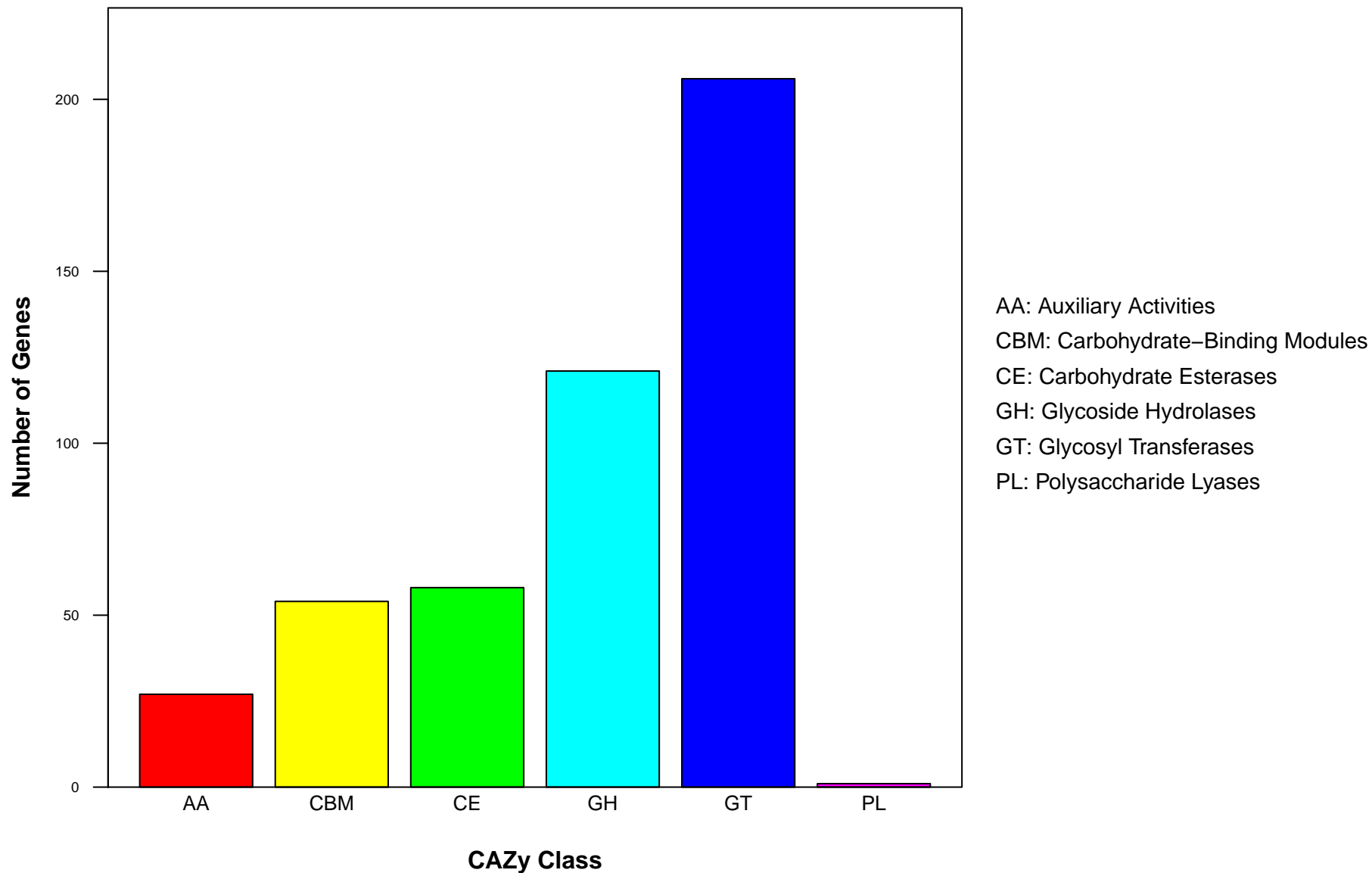

Supplement: Supplementary file 1 [file DataSheet_1.zip › Figure S6.pdf]

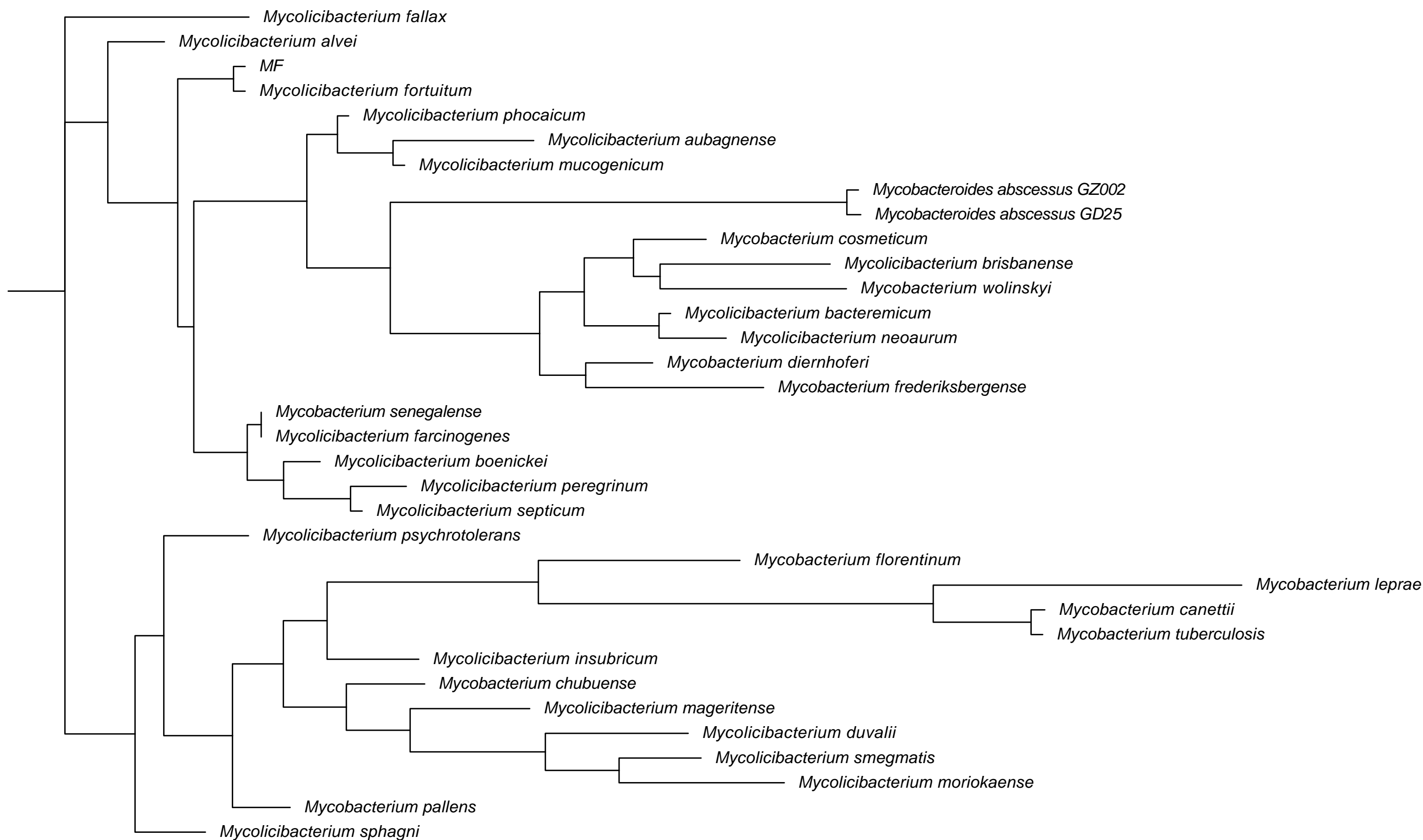

0.006

Supplement: Supplementary file 1 [file DataSheet_1.zip › Figure S8 (A).pdf]

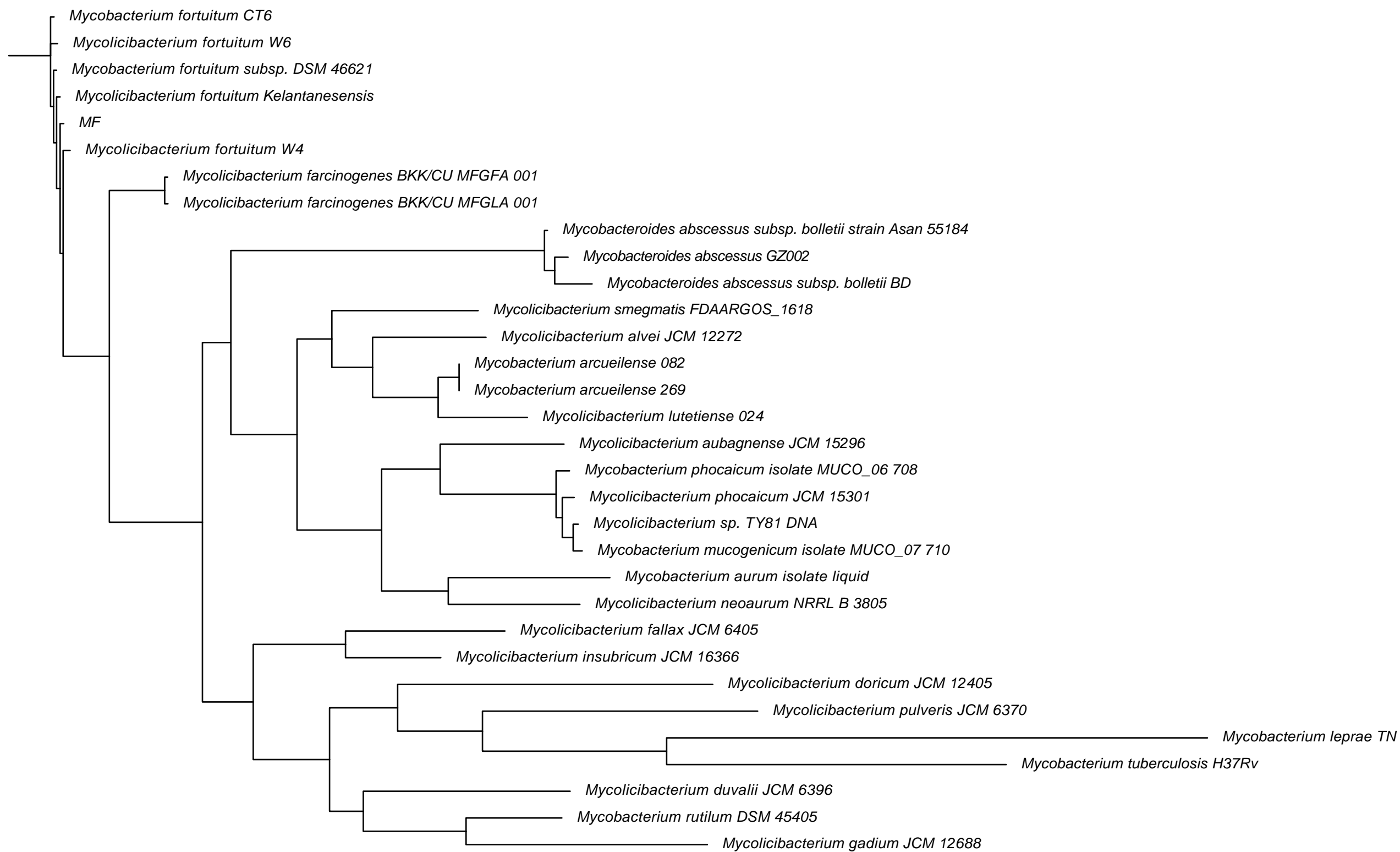

0.03

Supplement: Supplementary file 1 [file DataSheet_1.zip › Figure S8 (B).pdf]

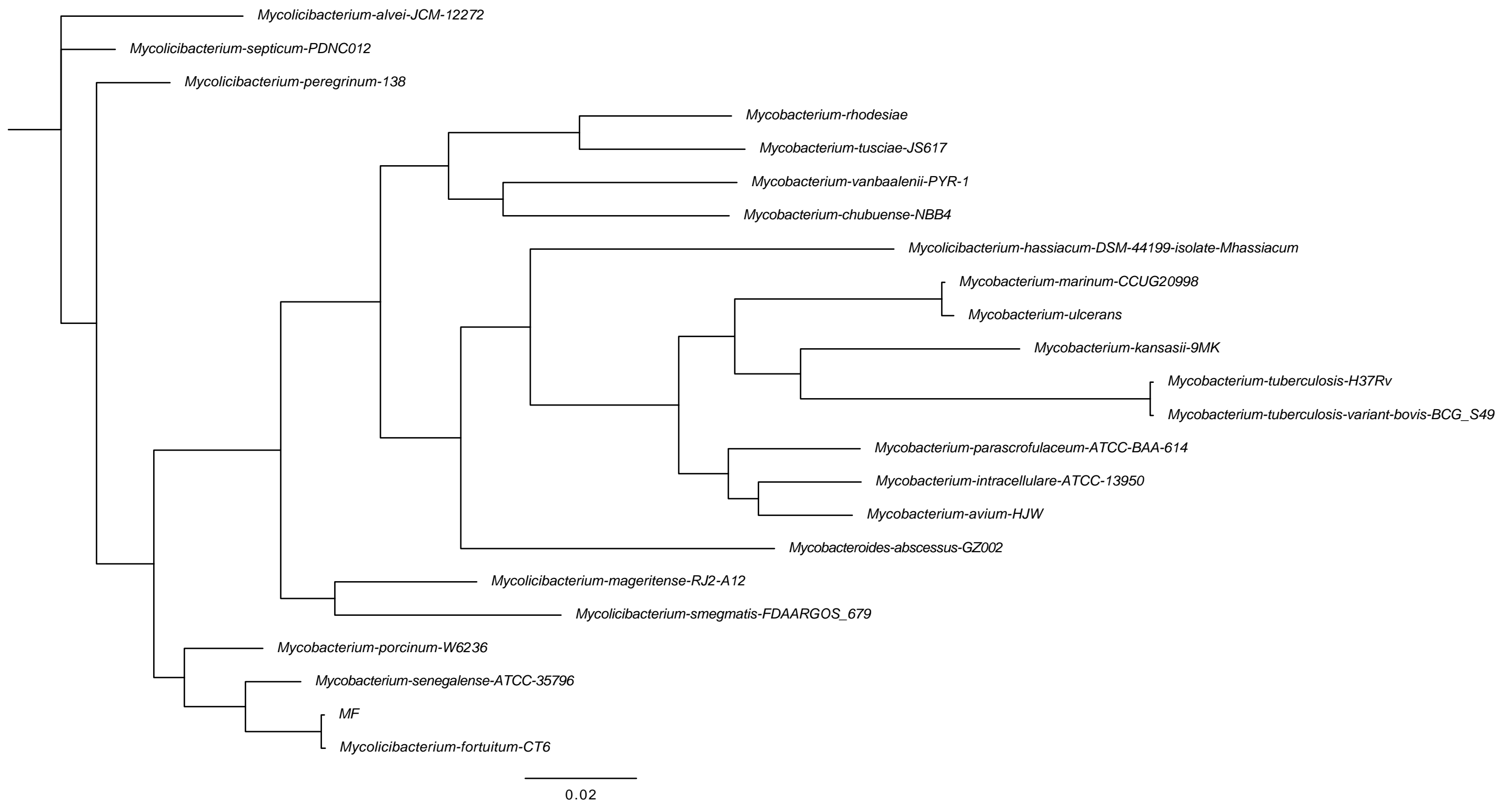

Supplement: Supplementary file 1 [file DataSheet_1.zip › Figure S8 (C).pdf]
